# Ectopic targeting of CG DNA methylation in Arabidopsis with the bacterial SssI methyltransferase

**DOI:** 10.1038/s41467-021-23346-y

**Published:** 2021-05-25

**Authors:** Wanlu Liu, Javier Gallego-Bartolomé, Yuxing Zhou, Zhenhui Zhong, Ming Wang, Somsakul Pop Wongpalee, Jason Gardiner, Suhua Feng, Peggy Hsuanyu Kuo, Steven E. Jacobsen

**Affiliations:** 1 Department of Orthopedic of the Second Affiliated Hospital of Zhejiang University School of Medicine, Zhejiang University, Hangzhou, China; 2 Zhejiang University-University of Edinburgh Institute (ZJU-UoE Institute), Zhejiang University School of Medicine, International Campus, Zhejiang University, Haining, China; 3 Department of Molecular, Cell and Developmental Biology, University of California at Los Angeles, Los Angeles, CA USA; 4 Instituto de Biología Molecular y Celular de Plantas (IBMCP), CSIC-Universidad Politécnica de Valencia, Valencia, Spain; 5 Department of Microbiology, Faculty of Medicine, Chiang Mai University, Chiang Mai, Thailand; 6 Eli & Edythe Broad Center of Regenerative Medicine & Stem Cell Research, University of California at Los Angeles, Los Angeles, CA USA; 7 Howard Hughes Medical Institute, University of California at Los Angeles, Los Angeles, CA USA

**Keywords:** Molecular engineering in plants, DNA methylation, Plant molecular biology

## Abstract

The ability to target epigenetic marks like DNA methylation to specific loci is important in both basic research and in crop plant engineering. However, heritability of targeted DNA methylation, how it impacts gene expression, and which epigenetic features are required for proper establishment are mostly unknown. Here, we show that targeting the CG-specific methyltransferase M.SssI with an artificial zinc finger protein can establish heritable CG methylation and silencing of a targeted locus in *Arabidopsis*. In addition, we observe highly heritable widespread ectopic CG methylation mainly over euchromatic regions. This hypermethylation shows little effect on transcription while it triggers a mild but significant reduction in the accumulation of H2A.Z and H3K27me3. Moreover, ectopic methylation occurs preferentially at less open chromatin that lacks positive histone marks. These results outline general principles of the heritability and interaction of CG methylation with other epigenomic features that should help guide future efforts to engineer epigenomes.

## Abstract (web summary)

The ability to target DNA methylation to specific loci is important for both basic and applied research. Here, the authors fuse CG-specific methyltransferase SssI with an artificial zinc finger protein for DNA methylation targeting and show the chromatin features favorable for efficient gain of methylation.

## Introduction

**Fig. 1:**
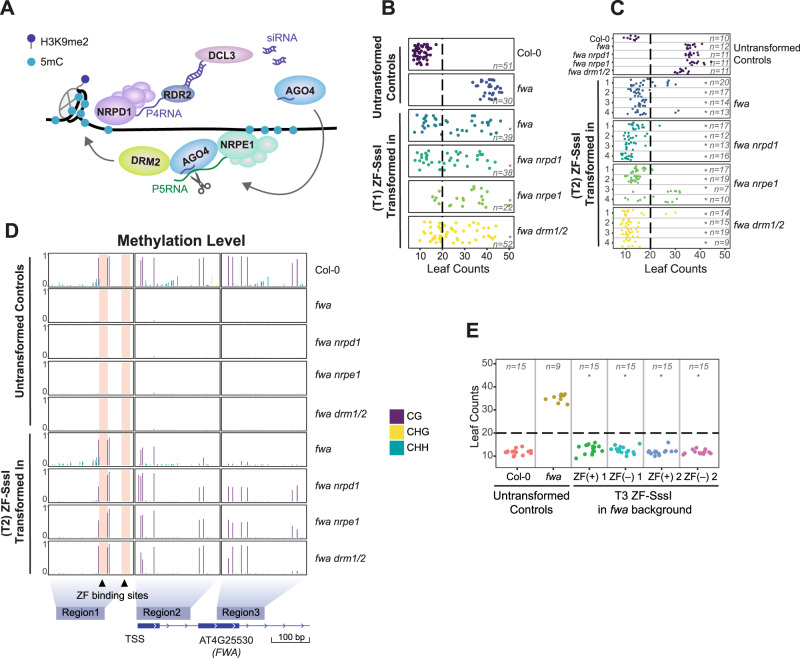
SssI-targeted methylation at the *FWA* promoter cause silencing. **A** Cartoon depicting the RNA-directed DNA methylation pathway. **B** Flowering time of untransformed controls, and T1 ZF-SssI lines in the *fwa* background or mutants that have been introgressed into the *fwa* background. The *X*-axis represents the number of leaves. Each dot represents one individual plant. Dashed lines indicate the cutoff we chose to define ‘early flowering’ versus ‘late flowering’ (*p-value < 0.01 compared to *fwa*, Welch two-sample *t-*test). **C** Flowering time of untransformed control lines and four representative ZF-SssI T2 lines in the *fwa* background or mutants that have been introgressed into the *fwa* background (*p-value < 0.01 compared to relative controls, Welch two-sample *t*-test). **D** CG, CHG, and CHH methylation levels over the *FWA* promoter measured by bisulfite (BS)-PCR-seq in untransformed controls and ZF-SssI in the *fwa* background or mutants that have been introgressed into the *fwa* background. The barplot represents data from one representative T2 plant for each genotype tested. Every single bar represents one cytosine. Black triangles and orange shaded rectangle regions indicate the designed ZF binding sites. The relative position of the three regions analyzed in the *FWA* gene are indicated as blue squares. **E** Flowering time of untransformed Col-0, *fwa*, and two representative T3 ZF-SssI lines with (+) or without (‒) the transgene. (* *p-*value < 0.01 compared to *fwa*, Welch two-sample *t*-test). Source data underlying Figs. 1B, 1C, and 1E are provided as a Source Data file.

DNA methylation is an evolutionarily conserved epigenetic modification that plays critical roles in silencing transposable elements and in regulating gene expression^1–3^. In *Arabidopsis*, DNA methylation occurs within three sequence contexts: CG, CHG, and CHH (where H represents A, T, or C)^4^. The establishment of DNA methylation in plants involves the DNA methyltransferase DOMAINS REARRANGED METHYLTRANSFERASE 2 (DRM2, the homolog of mammalian DNA methyltransferase DNMT3) through the plant-specific RNA-directed DNA Methylation (RdDM) pathway (Fig. 1A). RdDM involves the transcription of 30–40 nucleotide (nt) single-stranded RNAs (P4RNA)^5–7^ by RNA polymerase IV (Pol IV) that are then reverse transcribed by RNA-DEPENDENT RNA POLYMERASE 2 (RDR2)^8,9^, processed into 24 nt small interfering RNAs (siRNA) through DICER-LIKE 3 (DCL3)^10^, and loaded into ARGONAUTE 4 (AGO4)^11–13^. Next, siRNA-bound AGO4 recognizes noncoding P5RNAs, transcribed by Pol V, through sequence complementation^14^, which leads to their co-transcriptional slicing and triggers the recruitment of DRM2 and de novo methylation^15,16^ (Fig. 1A). After establishment, the maintenance of CG methylation requires the DNA methyltransferase METHYLTRANSFERASE 1 (MET1, the homolog of mammalian DNMT1), while maintenance of CHG and CHH methylation redundantly requires CHROMOMETHYLASE 3 (CMT3), CMT2, and DRM2^4,17,18^.

Symmetric CG methylation is conserved across different organisms and is distributed mostly over heterochromatic or genic areas^3,19^. In plants, methylation over heterochromatic regions occurs in CG, CHG, and CHH contexts and plays an important role in transcriptional silencing of transposable elements and repetitive sequences^4,20^. By contrast, methylation over genic regions, known as gene body methylation (gbM), occurs specifically in the CG context, positively correlates with gene expression, and is enriched over constitutively expressed genes^21,22^. Despite its high degree of conservation across organisms, the function of gbM is not well understood^23^. Studies in different organisms have proposed various functions for gbM including regulation of gene expression, alternative splicing, antisense transcription, enhancement of splicing accuracy through exon definition, inhibition of RNA Polymerase II (Pol II) initiation, and reduction of Pol II elongation efficiency^3,24–27^. However, most studies in different plant species and natural accessions of the model plant *Arabidopsis* that present differences in gbM levels have shown a limited effect of this modification on gene expression^28–30^. Similarly, gbM does not seem to affect the overall pattern of different histone modifications in plants^29^. An exception is the histone variant H2A.Z, where gbM has been hypothesized to prevent H2A.Z expansion into gene bodies and transcription of aberrant transcripts^21,31^. However, a more recent study did not find a connection between H2A.Z and gbM in *Arabidopsis* and *E. salsugineum*^29^, making this connection somewhat controversial. Moreover, the H3K27me3 modification, which accumulates over tissue-specific or developmental genes, has also been shown to anticorrelate with gbM in *Arabidopsis*^32^.

With the development of DNA targeting tools such as artificial zinc fingers (ZF), TAL effectors, and CRISPR/Cas9 systems, controlled manipulation of DNA methylation at specific genomic loci has been successfully achieved in plants and animals^33–39^. Our recent study in *Arabidopsis* showed that targeting of different RdDM components tethered by an artificial ZF protein is sufficient to target methylation to ZF binding sites in the genome^34^. Previous studies in different organisms have used the *Spiroplasma* sp. strain MQ1 CG methyltransferase M.SssI (SssI) to target methylation^39,40^.

In this work, we fuse SssI to an artificial ZF designed to target the *FLOWERING WAGENINGEN* (*FWA)* promoter^33,34,38^ (ZF-SssI) and test its ability to target methylation in *Arabidopsis*. The ZF-SssI fusion protein successfully targets heritable DNA methylation to the *FWA* promoter and other ZF off-target sites. Moreover, ZF-SssI plants exhibit genome-wide ectopic CG methylation, especially over exons and transcription termination sites (TTS), suggesting nonspecific ectopic activity of the ZF-SssI fusion. Importantly, ectopic CG methylation is highly heritable over most genomic regions. We leverage this system to study features that characterize loci ectopically methylated by ZF-SssI and the effect that the addition of CG methylation has on gene expression, histone modifications, and histone variants.

## Results

### SssI-targeted methylation at the *FWA* promoter causes silencing

To test whether SssI is capable of targeting CG DNA methylation in *Arabidopsis*, we fused SssI with a previously described artificial zinc finger (ZF) designed to target the *FWA* promoter^33^. *FWA* is normally repressed by DNA methylation over its promoter region in wild-type Col-0 plants and the loss of DNA methylation induces heritable *fwa* epialleles with ectopic expression of the *FWA* gene causing a late flowering phenotype^41^. When expressed in plants harboring an unmethylated *fwa* epiallele, ZF-SssI successfully targeted methylation to the *FWA* promoter, silencing *FWA* and triggering a change from late to early flowering in the first generation of transformed plants (the T1 generation) (Fig. 1B–D and Supplementary Fig. 1). As expected, ZF-SssI plants showed high levels of CG methylation at *FWA*. ZF-SssI plants also showed restored, but lower levels of CHH methylation at *FWA* compared to untransformed control plants (Fig. 1D). The partial restoration of CHH methylation is most likely the result of CG-methylation-dependent recruitment of the methyl-DNA binding proteins SU(VAR)3-9 homologs SUVH2 and SUVH9, which in turn recruit RdDM activity and the observed CHH methylation^33,42^.

To study if ZF-SssI mediated methylation and silencing are dependent on the RdDM pathway, we transformed ZF-SssI into the *fwa nrpd1* (*NRPD1* encodes the catalytic subunit of Pol IV, Fig. 1A), *fwa nrpe1* (*NRPE1* encodes the catalytic subunit of Pol V, Fig. 1A), and *fwa drm1 drm2 (fwa drm1/2)* backgrounds (DRM1 is a lowly expressed DRM2 paralog^43^, Fig. 1A). ZF-SssI successfully established methylation and silencing of *FWA* in all three mutant backgrounds (Fig. 1B–D and Supplementary Fig. 1) indicating that ZF-SssI can methylate the *FWA* promoter independently of the RdDM pathway and, consistent with previous reports, that promoter CG methylation is sufficient to silence *FWA*^34^. Moreover, targeted methylation in these backgrounds was depleted of CHH methylation consistent with the idea that the CHH methylation found in ZF-SssI lines in the *fwa* background was due to the recruitment of RdDM by targeted CG methylation (Fig. 1D).

Targeted methylation was previously shown to be heritable after segregating away the effector construct^33–35^. To investigate the heritability of targeted CG methylation, we segregated away the ZF-SssI transgene in the T3 generation. We observed an early flowering phenotype in two independent T3 ZF-SssI lines either with or without the transgene, indicating that the targeted CG methylation and *FWA* silencing were heritable (Fig. 1E).

### ZF-SssI expression caused targeted and nontargeted ectopic methylation genome-wide

**Fig. 2:**
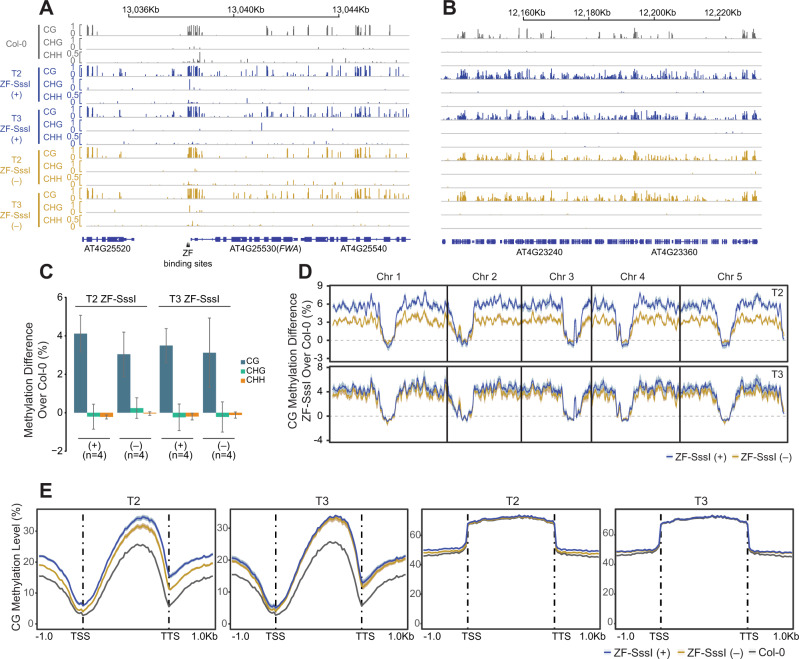
SssI-targeted genome-wide heritable CG methylation. **A** Screenshot for CG, CHG, and CHH methylation of representative ZF-SssI lines during T2 and T3 with (+) or without (‒) the transgene in Col-0 background over the *FWA* locus. Each bar represents a single base-pair. Black triangles indicate the designed ZF binding sites. **B** Screenshot for CG, CHG, and CHH methylation of representative ZF-SssI lines during T2 and T3 with (+) or without (‒) the transgene in the Col-0 background over a selected genomic region. **C** Barplot of genome-wide CG, CHG, and CHH methylation difference for ZF-SssI lines during T2 and T3 with (+) or without (‒) the transgene over Col-0. Error bars represent standard errors, center of error bars represent mean. **D** Genome-wide metaplot of CG methylation for ZF-SssI lines during T2 and T3 with (+) or without (‒) the transgene. The curve represents the mean, shaded area around the curve represents standard errors (*n* = 4). **E** CG methylation metaplot for Col-0 and ZF-SssI lines during T2 and T3 with (+) or without (‒) the transgene over protein-coding genes (left two panels) or transposable elements (TEs) (right two panels). The curve represents the mean, shaded area around the curve represents standard errors (*n* = 4).

While the ZF was originally designed to bind to two tandem repeats in the *FWA* promoter (Fig. 1D), our recent study showed that ZF-RdDM fusions bind to thousands of ‘off-targets’ in the genome resulting in hundreds of hypermethylated loci^34^. Therefore, we analyzed genome-wide CG methylation levels in the ZF-SssI lines. Since the *fwa* epiallele used was generated by crossing wild-type Col-0 with *met1* mutant plants and, thus, contains a chimeric epigenetic landscape^34^, we instead transformed ZF-SssI into wild-type Col-0 plants for this experiment. We performed whole-genome bisulfite sequencing (WGBS) in two biological replicates of two independent ZF-SssI transgenic lines in T2 and T3 generations with the transgene present (+) or segregated away (‒) (*n* = 16 in total, Supplementary Data 1). Browsing the tracks generated from the WGBS clearly showed widespread ectopic CG methylation (but not CHG or CHH methylation) over different genomic regions in ZF-SssI (+) or ZF-SssI (‒) lines during T2 and T3 (Fig. 2A, B, and Supplementary Fig. 2A, B). On average, we observed around 3–4% genome-wide hyper CG methylation (hCG) mainly over euchromatic regions in ZF-SssI lines compared to Col-0 (Fig. 2C, D). Consistent with the observation of genome-wide hCG over euchromatic regions, we observed hCG over protein-coding genes (Fig. 2E). By contrast, no hCG was detected over transposable elements (TEs) (Fig. 2E).

To test whether the observed hCG was due to direct ZF-SssI binding, we performed chromatin immunoprecipitation sequencing (ChIP-seq) to map the genome-wide binding sites of ZF-SssI. Some hypermethylated regions were bound by the ZF-SssI, while many other hCG regions did not have a ZF peak (Supplementary Fig. 3A). ChIP-seq results identified 2151 ZF-SssI binding sites. Consistent with previous results from other ZF-RdDM fusions^34^, the ZF-SssI showed a preference for promoter regions and preferentially bound to the core cis-motif sequence specified by the inner ZF repeats (Supplementary Fig. 3B, C). Analysis of ZF-SssI ChIP-seq and WGBS showed a clear hCG enrichment over ZF-SssI binding sites but also showed random hCG flanking the ZF-SssI summits (Supplementary Fig. 3D). This suggests that the hCG we detected is a combination of ZF-SssI-targeted methylation as well as nonspecific ectopic methylation triggered by ZF-SssI.

### Hyper CG-methylated sites in ZF-SssI occur preferentially over previously unmethylated gene bodies

**Fig. 3:**
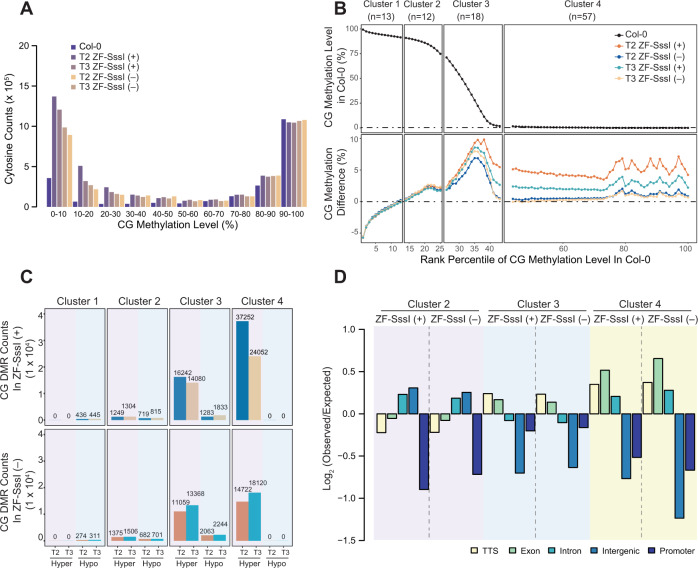
Characteristics of hyper CG-methylated sites in ZF-SssI. **A** CG cytosine counts grouped by CG methylation level in Col-0 and ZF-SssI lines during T2 and T3 with (+) or without (‒) the transgene. **B** CG methylation level of 200 bp bins in Col-0 (upper panel) and CG methylation difference in ZF-SssI lines during T2 and T3 with (+) or without (‒) the transgene (lower panel) ranked by the CG methylation level in Col-0. Methylation difference is the absolute difference in CG methylation levels between ZF-SssI lines and Col-0. Four clusters are defined by the methylation level of 200 bp bins in Col-0. N represents the number of ranked percentiles within the cluster. **C** Count of hyper and hypo differentially methylated regions (DMRs) in ZF-SssI lines during T2 and T3 with (+) or without (‒) the transgene in the four clusters. **D** Genomic distribution (Observed versus Expected) of the hyper CG DMRs in ZF-SssI lines during T2 and T3 with (+) or without (‒) the transgene in Clusters 2, 3, and 4.

We further investigated the ectopic CG methylation caused by ZF-SssI to (i) analyze the characteristics of the CG sites that are competent to become hypermethylated, (ii) study the heritability of hCG sites, and (iii) investigate the potential crosstalk between hCG, gene expression, and histone modifications. We first analyzed the cytosine counts in Col-0 and ZF-SssI lines with different levels of CG methylation. In general, ZF-SssI lines showed an overall increase in methylated CG sites compared to Col-0, except for CG sites with saturated (90–100%) methylation levels in Col-0 (Fig. 3A).

To visualize this in more detail, we examined the relationship between pre-existing CG methylation and ZF-SssI-dependent hCG. We rank-ordered the CG methylation in 200 bp bins in Col-0 and plotted this along with the difference in CG, CHG, and CHH methylation levels between ZF-SssI lines and Col-0 (Fig. 3B and Supplementary Fig. 4A, B). After removing bins with no CG methylation in any of the genotypes (Col-0, ZF-SssI (+) or ZF-SssI (‒)), we divided the remaining 200 bp bins into four clusters based on the CG, CHG, and CHH methylation levels in Col-0 and the CG methylation difference between ZF-SssI lines and Col-0 (Fig. 3B and Supplementary Fig. 4A, B). Cluster 1 contained bins with high pre-existing CG, CHG, and CHH methylation in Col-0, and hypo CG methylation in ZF-SssI lines compared to Col-0 (top 13 ranked percentiles). Cluster 2 contained bins with pre-existing CG, CHG, and CHH methylation in Col-0 and hCG in ZF-SssI (ranked percentiles 14–25). Cluster 3 contained bins with pre-existing CG methylation but no CHG or CHH methylation in Col-0, and hCG in ZF-SssI (ranked percentiles 26–43). Cluster 4 showed no pre-existing DNA methylation in Col-0 but hCG in ZF-SssI (ranked percentiles 44–100). Consistent with the genome-wide methylation analysis (Fig. 2C), no hyper CHG or hyper CHH methylation was observed in Clusters 1–4 (Supplementary Fig. 4A, B). Using the 200 bp regions from these four clusters, we defined differentially methylated regions (DMRs) comparing ZF-SssI (+) or ZF-SssI (‒) lines during T2 and T3 with Col-0 for each cluster. In general, we observed a significant overlap of hypermethylated CG DMRs (hCG DMRs) between ZF-SssI lines during T2 and T3 (Supplementary Fig. 4C). To include all the potential hCG sites, we utilized the union set of T2 and T3 hCG DMRs for further analyses. Consistent with Fig. 3B, DMR analysis indicated that Cluster 1 only presented hypomethylated DMRs, while Clusters 2–4 showed more hypermethylated CG DMRs than hypomethylated DMRs in both ZF-SssI (+) and ZF-SssI (‒) lines (Fig. 3C). The DMR analysis was therefore consistent with the analysis of overall levels of methylation in these clusters.

We focused further analyses on the clusters showing hCG DMRs (2, 3, and 4; Supplementary Data 2). To have a control set with comparable pre-existing CG methylation in Col-0, we generated CG-methylation-equivalent (mCG-equivalent) control regions with the same number of 200 bp bins randomly selected from the same ranked percentile of the hCG DMRs. Sequence context analysis in Clusters 2, 3, and 4 showed that hCG DMRs in ZF-SssI lines have a significant preference for regions with higher (C + G) percentage and CG, CHG, and CHH densities compared to mCG-equivalent control regions (Supplementary Fig. 4D, E). We also studied the genomic distribution of hCG DMRs in Clusters 2, 3, and 4. Compared to mCG-equivalent control regions, hCG DMRs in Cluster 2 were enriched in introns and intergenic regions and depleted mostly in promoters (Fig. 3D). By contrast Clusters 3 and 4 were mainly enriched over transcription termination sites (TTS) and exons, and were depleted over intergenic and promoter regions (Fig. 3D). This profile is different than that observed for the ZF-SssI binding distribution (Supplementary Fig. 3B) and supports the idea that a fraction of the hCG observed is independent of ZF binding to chromatin. In summary, hypermethylated regions were mostly enriched over gene bodies and showed a preference for higher (C + G) percentage and CG, CHG, and CHH densities. Moreover, this analysis highlights the recalcitrant nature of promoters for gaining ectopic methylation.

### Hyper CG DMRs occur preferentially at relatively less accessible chromatin

**Fig. 4:**
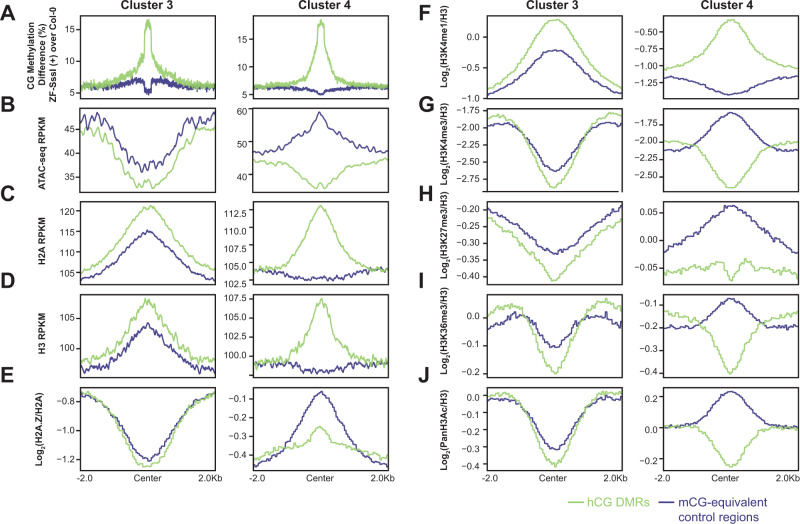
The epigenetic landscape for hyper CG DMRs of Clusters 3 and 4 in Col-0. Metaplot of the CG methylation difference of ZF-SssI over Col-0 (ZF-SssI (+) lines during T2 and T3 merged) (**A**), ATAC-seq signals (**B**), H2A (**C**) and H3 (**D**) occupancy, H2A.Z (**E**), H3K4me1 (**F**), H3K4me3 (**G**), H3K27me3 (**H**), H3K36me3 (**I**), and PanH3Ac enrichment (**J**) over hyper CG DMRs and CG-methylation-equivalent control regions in Clusters 3 and 4.

To investigate the epigenetic landscape of hCG sites, we performed ATAC-seq and ChIP-seq for various histone marks in Col-0. We plotted the CG methylation difference, ATAC-seq signal, H2A, H3, H2A.Z, H3K4me1, H3K4me3, H3K27me3, H3K36me3, and PanH3Ac (Fig. 4, Supplementary Data 3, and Supplementary Data 4) over hCG DMRs and mCG-equivalent control regions. For these analyses, we focused on Clusters 3 and 4 that have a much higher number of hyper DMRs than Cluster 2 (Fig. 3C) and represent regions with either pre-existing CG methylation (Cluster 3), or regions that gained CG methylation de novo (Cluster 4).

Compared to the surrounding chromatin, the landscape of hCG DMRs and mCG-equivalent control regions in Cluster 3 showed a similar trend with lower chromatin accessibility, higher levels of unmodified H3 and H2A histones and H3K4me1, and lower levels of H2A.Z, H3K4me3, H3K27me3, H3K36me3, and H3 acetylation (Fig. 4A–J). Chromatin over mCG-equivalent control regions in Cluster 4 showed a more open conformation and was enriched with activating marks (H3K4me3, H3K36me3, and H3 acetylation) as well as H2A.Z and H3K27me3 (Fig. 4B, E, G–J), while it was slightly depleted in unmodified H3 histones, H2A histones, and H3K4me1 (Fig. 4C, D, F). The hCG regions in Cluster 4 contained features that resembled those in Cluster 3, with higher levels of unmodified histones and H3K4me1 and lower chromatin accessibility and activating marks, except for the preference of higher H2A.Z levels (Fig. 4B–J). H2A.Z and H3K27me3 have been shown to anticorrelate with DNA methylation levels^31,32^. Consistent with this, hCG regions in Cluster 4 showed lower levels of these two marks when compared to control regions (Fig. 4E, H). In summary, we conclude that ZF-SssI-mediated ectopic methylation usually occurs over less accessible chromatin, is enriched in H3K4me1, and is associated with low levels of activating histone marks, H2A.Z, and H3K27me3.

### Genome-wide hCG triggers limited changes in gene expression

We used the ectopic gbM obtained with ZF-SssI to study the possible role of gbM in gene expression using RNA-seq (Supplementary Data 5). Although ZF-SssI lines showed high levels of ectopic CG methylation over hundreds of gene bodies, global gene expression levels were similar to Col-0 (Supplementary Fig. 5A). The number of differentially expressed genes (DEG) identified in ZF-SssI (+) or ZF-SssI (‒) lines during T2 and T3 compared to Col-0 was variable (Supplementary Fig. 5B and Supplementary Data 6). We detected 61 shared upregulated and 41 shared downregulated DEGs in ZF-SssI (+) lines (Supplementary Fig. 5C, E). ZF-SssI (‒) lines showed 10 shared downregulated DEGs while no common upregulated DEGs (Supplementary Fig. 5D, F). To test the association of hCG DMRs with DEGs, we analyzed our data with RAD^44^ (Region Associated DEGs, https://labw.org/rad) and found that hCG DMRs flanking 1 kb of the transcriptional start sites (TSS) significantly correlated with downregulated genes in ZF-SssI (+) with the highest correlation at the TSS (Supplementary Fig. 5G and Supplementary Data 7). This indicates a repressive role in transcription of the ectopic methylation located in regions proximal to the TSS. A similar trend was observed between hCG DMRs and DEGs in ZF-SssI (‒) lines, although this association was not significant (Supplementary Fig. 5H and Supplementary Data 7).

Gene body methylation has previously been linked to alternative splicing^24–26^. Thus, we analyzed the alternative splicing events in our RNA-seq dataset (Supplementary Fig. 6A). With rMATs^45^, we analyzed skipped exon (SE), alternative 5ʹ splice site (A5SS), alternative 3ʹ splice site (A3SS), mutually exclusive exons (MXE), and retained intron (RI) for all protein-coding genes. Comparing to Col-0, we indeed observed some alternative splicing events (Supplementary Fig. 6A). However, the increased alternative splicing events (Inclusion level > 0) in ZF-SssI were comparable to those in Col-0 (Inclusion level < 0). Thus, we detected little correlation between hCG and alternative splicing events.

To test whether there were more localized differences in gene expression level over genes with ectopic gbM, we classified genes into ‘De novo gbM’ and ‘Enhanced gbM’ groups. We defined genes with less than 3% CG methylation and more than 10% CG methylation enhancement as ‘De novo gbM’ genes, and those with more than 3% CG methylation and more than 10% CG methylation enhancement as ‘Enhanced gbM’ genes (Supplementary Fig. 6B and Supplementary Data 8). Metaplot analysis of ZF-SssI RNA-seq data over ‘De novo gbM’ and ‘Enhanced gbM’ groups indicated limited changes of gene expression levels in both groups (Supplementary Fig. 6C). Moreover, we observed very few up- or downregulated DEGs in ZF-SssI (+) that overlapped with ‘De novo gbM’ and ‘Enhanced gbM’ genes (Supplementary Fig. 6D). Therefore, consistent with previous studies using *Arabidopsis* epiRILs and other flowering plant species that lack gbM^28–30^, our analyses suggest a limited role for gbM in transcriptional regulation.

### Gain of methylation at gene bodies reduces H2A.Z and H3K27me3 accumulation

**Fig. 5:**
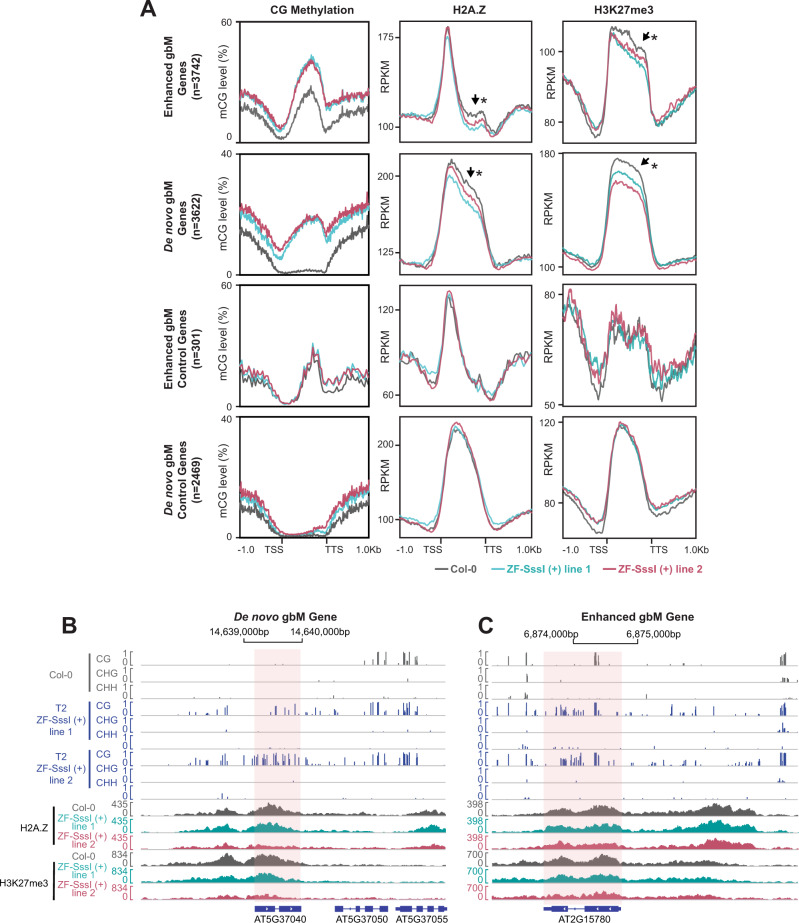
H2A.Z and H3K27me3 reduction over hypermethylated genes. **A** Metaplots of CG methylation (T2 and T3 merged), H2A.Z, and H3K27me3 levels in Col-0 and two representative ZF-SssI (+) lines over genes with ‘Enhanced gbM’ or ‘De novo gbM’ as well as a set of control genes with similar gene body CG methylation but no hypermethylation. *p-value < 0.05, Welch Two-Sample *t*-test (‘De novo gbM’ group, hypothesis testing performed on full-length genic regions; ‘Enhanced gbM’ group, hypothesis testing performed on last 50% of genic regions). The black arrow indicates the regions with reduced H2A.Z and H3K27me3. **B, C** Screenshot of WGBS tracks in Col-0 and two representative T2 ZF-SssI (+) lines, as well as H2A.Z and H3K27me3 ChIP-seq tracks in Col-0 and two representative T2 ZF-SssI (+) lines over selected ‘De novo gbM’ (**B**) and ‘Enhanced gbM’ (**C**) genes. Source data underlying Figure 5A are provided as a Source Data file.

The gain of gbM in the ZF-SssI lines might disturb the distribution pattern of histone modifications and chromatin accessibility within gene bodies. To test this, we performed additional ATAC-seq and ChIP-seq experiments for different histone marks in ZF-SssI lines and compared this to the signal obtained for Col-0 controls. For these analyses we focused on the sets of ‘Enhanced gbM’ and ‘De novo gbM’ genes previously described (Supplementary Fig. 6B). In addition, we defined a set of control genes for the ‘Enhanced gbM’ and ‘De novo gbM’ groups with similar CG methylation levels in Col-0 but no hCG in the ZF-SssI lines. No significant differences were observed for the different marks tested over both ‘Enhanced gbM’ and ‘De novo gbM’ genes except for H2A.Z and H3K27me3 (Fig. 5, Supplementary Fig. 7, and Supplementary Fig. 8A, B). The H2A.Z histone variant showed two different profiles over genes with ‘Enhanced gbM’ and ‘De novo gbM’ groups (Fig. 5A). In the ‘Enhanced gbM’ group, the H2A.Z signal was more prominent over the TSS, which is characteristic of methylated genes with medium- to high expression^31,46,47^ (Fig. 5A). In the ‘De novo gbM’ group, the distribution of the H2A.Z signal was more even over gene bodies. According to previous reports, this distribution is characteristic of lowly expressed genes and it usually overlaps with the silencing mark H3K27me3^32^ (Fig. 5A). Interestingly, H2A.Z signal was lower at both groups of genes in the ZF-SssI lines (Fig. 5A–C, Supplementary Fig. 8A, B, and Supplementary Data 9). The ‘Enhanced gbM’ group showed a reduction over the second half of the gene body region while the ‘De novo gbM’ group presented a reduction over most of the gene body (Fig. 5A). H2A.Z signal over these regions in the control set of genes was similar in ZF-SssI lines and Col-0 (Fig. 5A). In the case of the H3K27me3 mark, we observed a reduction in ZF-SssI lines over most of the gene body for both ‘Enhanced gbM’ and ‘De novo gbM’ groups, while signal over control regions was not affected (Fig. 5A–C, Supplementary Fig. 8A, B, and Supplementary Data 9). Interestingly, the ‘Enhanced gbM’ group showed high levels of the H3K27me3 mark, which is unexpected considering the reported negative correlation between the accumulation of gene body methylation and H3K27me3 mark^32^ (Fig. 5A, C). Thus, we separated ‘Enhanced gbM’ genes into genes with or without H3K27me3 (Supplementary Fig. 8C-E). As expected, this analysis revealed a subset of ‘Enhanced gbM’ genes with no H3K27me3 (Supplementary Fig. 8C). These genes accumulated H2A.Z, mostly around the TSS, and consistent with our previous analyzes (Fig. 5A), showed a decrease in this mark over the second half of the gene body in the ZF-SssI lines (Supplementary Fig. 8D). Interestingly, this analysis revealed a smaller subset of genes from the ‘Enhanced gbM’ group that contained low levels of pre-existing gbM and H3K27me3. In line with the previous analysis, H3K27me3 and H2A.Z signals were reduced in these genes over the whole coding region in the ZF-SssI lines (Supplementary Fig. 8D). We also divided the ‘De novo gbM’ group into genes with or without H3K27me3, which resulted in similar conclusions (Supplementary Fig. 8E). Moreover, we observed higher ectopic gene body CG methylation levels in ‘De novo gbM’ genes without H3K27me3 compared to ‘De novo gbM’ genes with H3K27me3 (Supplementary Fig. 8E). Together, these results are in line with previous reports^31,32^ and suggest that gbM has a negative effect on H2A.Z and H3K27me3 accumulation.

### Ectopic CG methylation is highly heritable

**Fig. 6:**
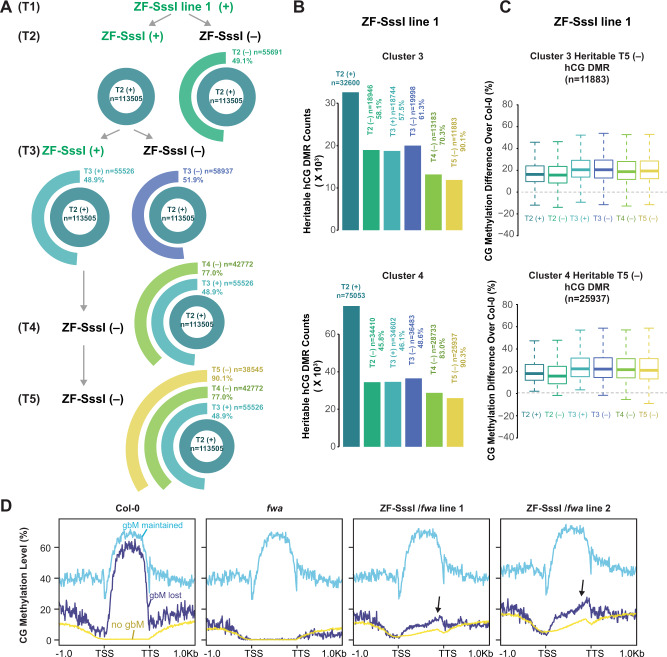
Targeted CG methylation is heritable. **A** Multilevel pie chart of the number of heritable hyper CG DMRs in ZF-SssI line 1 during T2, T3, T4, and T5 ((+) indicates ZF-SssI lines containing the transgene, which could be homozygous or heterozygous in the indicated generation; (‒) indicates plants without the transgene in the indicated generation). **B** Barplot for the number of heritable hyper CG DMRs over Clusters 3 and 4 in ZF-SssI line 1 during T2, T3, T4, and T5. For the percentage of heritable hyper CG DMRs T2 (‒), T3 (+), and T3 (‒) are compared with T2 (+); T4 (‒) is compared with T3 (+); T5 (‒) is compared with T4 (‒). **C** Boxplot of CG methylation difference level of ZF-SssI during T2 to T5 over Col-0 in Clusters 3, and 4 heritable hyper CG DMRs in ZF-SssI line 1 (‒) during T5. The middle line shows the median; boxes represent the 25th (bottom) and 75th (top) percentiles; and bars represent the minimum and maximum points within the 1.5× interquartile range. **D** Metaplot of CG methylation in Col-0, *fwa*, and two ZF-SssI lines in the *fwa* background over ‘no gbM’, ‘gbM lost’, and ‘gbM maintained’ protein-coding genes. ‘no gbM’ represents genes with no gbM in *fwa* and Col-0; ‘gbM lost’ represents genes that lost gbM in *fwa* compared with Col-0; ‘gbM maintained’ represents genes that maintained gbM in *fwa* compared to Col-0. The black arrow indicates the hCG in ZF-SssI/*fwa* lines over genes that had lost gbM in *fwa*.

We next examined in more detail the heritability of ectopic CG methylation in ZF-SssI plants where the transgene had been segregated away for two (T5 (‒)) generations and compared this with methylation levels in lines that had lost the transgene for one generation (T2 (‒), T3 (‒), and T4 (‒)) (Fig. 6A and Supplementary Fig. 9A, B). Comparing T4 (‒) and T5 (‒) plants, the targeted CG methylation was faithfully maintained with around 2–3% of genome-wide hCG methylation, mainly over euchromatic regions, compared to their side-by-side Col-0 controls (Supplementary Fig. 9C, D). Consistent with what we observed in T2 and T3 generations (Fig. 2E), we observed hCG over protein-coding gene bodies but not transposable elements (Supplementary Fig. 9E, F).

In order to quantify the heritability of targeted hCG, we defined hCG DMRs comparing T4 and T5 ZF-SssI (‒) with their corresponding Col-0 controls and calculated the percentage of heritable hCG DMRs across multiple generations (from T2 to T5). In both transgenic lines used in this study, we observed a consistently high percentage (around 50–90% in the last generation) of heritable hCG DMRs (Fig. 6A and Supplementary Fig. 10A) indicating that ZF-SssI-dependent CG methylation is highly heritable. We then calculated the overlap between hCG DMRs of T2 to T5 generations in Clusters 3 and 4. Comparing heritable hCG DMRs in T2 ZF-SssI (‒) with T2 ZF-SssI (+), we identified 39-58% heritable hCG DMRs in Clusters 3 and 4 for both transgenic lines (Fig. 6B and Supplementary Fig. 10B). Similarly, 37–67% of hCG DMRs were heritable when comparing T3 ZF-SssI (either (+) or (‒)) with T2 ZF-SssI (+) (Fig. 6B and Supplementary Fig. 10B). A high level of heritable hCG DMRs (64–83%) was observed when comparing T4 ZF-SssI (‒) with T3 ZF-SssI (+) in Clusters 3 and 4. Most of the hCG DMRs (72-90%) were maintained when comparing T5 to T4 ZF-SssI (‒) plants (Fig. 6B and Supplementary Fig. 10B). Moreover, when we plotted the CG methylation level over heritable hCG DMRs identified in T5 ZF-SssI (‒) plants, we observed similar levels across multiple generations in both Clusters 3 and 4 (Fig. 6C and Supplementary Fig. 10C). These results confirm that, unlike CHH methylation^34^, once CG methylation is established it can be efficiently maintained. It is not surprising that hCG DMRs in Cluster 3 are heritable as these are sites that contain pre-existing CG methylation indicating that MET1 is already maintaining some CG methylation at these sites. However, high heritability in Cluster 4 regions, which did not have pre-existing methylation, indicates that ZF-SssI-dependent hCG can be efficiently maintained even without the ZF-SssI transgene.

We also analyzed CG methylation in *fwa* epimutant plants expressing ZF-SssI so that we could compare the occurrence of ZF-SssI-dependent hCG over regions that did not have pre-existing methylation with regions that were naturally methylated in wild-type but had lost this methylation in the *fwa* background prior to the introduction of ZF-SssI. The *fwa* epiallele is the result of a cross between Col-0 and the *met1* mutant, and thus presents a chimeric methylation profile where some gene bodies remain unmethylated while others recover wild-type methylation levels. We first classified genes into three groups including genes with no gbM (genes with less than 1% CG gbM in Col-0 and *fwa*), genes that had lost gbM in *fwa* (genes with more than 40% CG gbM in Col-0 but less than 1% CG gbM in *fwa*), and genes that maintained gbM in *fwa* (genes with more than 40% CG gbM in both Col-0 and *fwa*). We observed higher hCG in ZF-SssI lines in the *fwa* background over both protein-coding genes and transposable elements that had lost gbM in *fwa* than genes and transposable elements with no pre-existing gbM (Fig. 6D and Supplementary Fig. 10D). This result suggests that compared to genes without pre-existing CG gbM, genes that naturally displayed gbM have a higher tendency to become methylated after gbM was lost.

## Discussion

In this study, we used the bacterial CG methyltransferase SssI fused to an artificial zinc finger protein to target CG methylation in *Arabidopsis*. The ZF-SssI fusion was able to establish heritable CG, CHG, and CHH methylation over the *FWA* promoter and cause *FWA* silencing^34^. In addition, ZF-SssI targeted methylation and triggered early flowering in strong RdDM mutant backgrounds (*nrpd1*, *nrpe1*, and *drm1/2*) indicating that targeted CG methylation is independent of RdDM activity and sufficient to silence *FWA*. While the targeted methylation we detected by BS-PCR-seq over the *FWA* promoter is likely sufficient to silence *FWA* expression, ectopic methylation over other *FWA* regions might also contribute to its repression. Additionally, we also observed hCG methylation across the genome. While part of this hypermethylation was due to the binding of ZF-SssI to off-target sites, we also detected genome-wide ectopic CG methylation. Even though the ZF we designed is found mostly over promoter regions, the ectopic methylation accumulated preferentially over regions with less accessible chromatin landscapes. One possibility to explain this disparity is that the ChIP is revealing stable interactions between the ZF-SssI and chromatin, while the ectopic hCG is the consequence of unstable hit-and-run interactions between the ZF-SssI and regions that are more prone to become methylated and maintained by MET1. The expression of either a ZF-SssI version where the ZF is mutated to prevent binding to DNA or the expression of free SssI protein would help clarify the contribution of the ZF in ZF-SssI to the genome-wide hCG observed in these plants. Thus, in order to optimize the use of SssI for locus-specific targeting approaches, a more specific targeting system with fewer off-target sites is required. In this regard, CRISPR-dCas9 technology fused to SssI probably represents the best approach^35,39^. Additionally, SssI could be also exploited to trigger global hCG and generate epialleles in different plant species and crops by overexpressing the free SssI protein.

Although we detected widespread genome-wide hCG, CHG and CHH methylation remained constant indicating that RdDM was not recruited despite the known ability of CG methylation to recruit this pathway. One potential explanation for this is that the hCG we detected was mostly located over gene bodies that contain active epigenetic marks associated with transcription, such as H3K4 methylation that would likely prevent RdDM recruitment/activity. Indeed, Pol IV recruitment through the histone reader SAWADEE HOMEODOMAIN 1 (SHH1) is prevented by H3K4me3^48^. Previously, some ectopic hypermethylation effects have been observed in *Arabidopsis* when targeting methylation using the de novo methyltransferase DRM2^35^. However, this was largely restricted to non-CG sequence contexts and was mostly not heritable probably due to poor maintenance by MET1^35^.

We took advantage of the genome-wide hCG caused by ZF-SssI to study the epigenetic landscape that is favorable for CG methylation establishment. Hypermethylated regions were enriched over gene bodies and showed a preference for higher (C + G) and CG, CHG, and CHH densities (Fig. 3 and Supplementary Fig. 4). This is consistent with a previous observation that CG methylation correlates with higher CG density^49^. In addition, hCG methylated sites usually occurred over less accessible chromatin that was depleted of activating marks like H3K4me3, as well as H2A.Z and H3K27me3 (Fig. 4). These results are consistent with the previous observations that the RdDM pathway is repelled by H3K4me3^34,48^ and that DNA methylation anticorrelates with H2A.Z and H3K27me3^31,32^, and identify a chromatin landscape that is favorable for targeted CG methylation. This should be taken into consideration when using this or similar technologies to target CG methylation in plants. Interestingly, the shape of the ectopic gene body methylation was similar to the endogenous gene body methylation (Figs. 2 and 5). A possible interpretation of this result is that initial ectopic methylation deposited by ZF-SssI over gene bodies is maintained or amplified by MET1 which, in turn, is influenced by epigenetic marks like histone modifications, that accumulate differentially across the gene body regions. For instance, there is a positive correlation between H3K4me1 and gene body methylation (Fig. 4)^48^ that could help explain this characteristic distribution. It is worth noting that ectopically expressed DNA methyltransferases, including SssI, in yeast, Drosophila, and mammalian cells, have been utilized for footprinting of open nucleosome-depleted regions^50–53^. While we observed that SssI-dependent methylation was enriched over less accessible chromatin, we reasoned this might be due to the fact that SssI-dependent methylation is maintained/amplified by MET1, which is more efficient or active over closed chromatin regions with higher nucleosome densities^54^. Another possibility is that SssI may efficiently access and methylate open chromatin regions but the methylation is then removed by DNA glycosylases like ROS1^55^.

Gene body methylation has been proposed to be involved in various aspects of gene expression regulation such as alternative splicing, transcription initiation, and elongation^3,24–27^. We did observe a repressive effect of hCG on transcription when it occurred near the TSS region (Supplementary Fig. 5G). However, we observed only limited changes of gene expression or alternative splicing events over genes that gained hCG over their gene bodies (Supplementary Fig. 6), which is consistent with results from previous studies in plants^29,30^. One possibility is that the level of targeted CG methylation in our ZF-SssI lines is under the threshold level necessary to induce any significant transcriptional changes. Alternatively, gene body methylation may simply be a consequence of other epigenetic processes, as has been recently suggested^56^. It is worth mentioning that compared to previous studies where the effect of gbM on gene expression was studied in hypomethylated loss-of-function mutants, like *met1*, our gain-of-function study analyzes the effect of gbM in genes that have not been previously exposed to gbM.

H2A.Z is a histone variant strongly enriched over unmethylated, active genes^46,47^, and has been shown to be anticorrelated with DNA methylation in both plants and mammals^31,57,58^. Loss of DNA methylation in *met1* mutants, or through pharmacological inhibition or knockdown of DNMTs in mammals, led to the gain of H2A.Z occupancy over hypomethylated regions^31,59^. This led to the proposal that gbM might be involved in stabilizing gene expression by excluding H2A.Z^21,31^. Thus, the effect of hCG on transcription might be unmasked if ZF-SssI plants were treated with different environmental stresses where a fast global transcriptional response is required to adapt to the new environment. However, a different study failed to detect H2A.Z changes in flowering plants lacking gbM^29^. Even though the effect is mild, our results support the first model where DNA methylation has a negative impact on H2A.Z accumulation (Fig. 5). Considering that we did not detect significant changes in gene expression over hCG genes, it is possible that the observed effects on H2A.Z levels may not be sufficient to alter the transcriptional output or that we failed to detect these changes via bulk-level RNA-seq technique^21^. Therefore, the relevance of the connection between gbM, H2A.Z, and transcription is presently unclear.

Maintenance of CG methylation is essential for epigenetic memory during gametogenesis and transgenerational inheritance in plants^60,61^. Analysis of the methylation landscape over multiple generations has demonstrated that CG methylation can be highly heritable^62^. Recently, we published a study in which we achieved targeted methylation using RdDM components tethered to a zinc finger protein. Although most of the targeted methylation was in a CHH context, only those regions that gained CG methylation became highly heritable in the absence of the triggering construct^34^. Consistent with these observations, ZF-SssI-dependent CG methylation was highly heritable in the absence of the transgene, even over regions with no pre-existing methylation (Fig. 6 and Supplementary Fig. 10). This strongly confirms that CG methylation is required and sufficient for methylation inheritance in plants. Interestingly, unmethylated regions in a *met1*/wild-type cross that were previously methylated showed a stronger gain in methylation in the presence of ZF-SssI (Fig. 6D and Supplementary Fig. 10D) suggesting that these naturally methylated regions have some properties that make them more prone to become methylated. Even though we observed highly heritable targeted hCG, there were a fraction of cytosines that lost methylation upon segregation of the ZF-SssI construct. Perhaps a longer exposure of these regions to ZF-SssI, or a free SssI protein, over multiple generations would promote the fixation of hCG, which would be needed in order to generate the maximum number of epialleles for breeding programs.

In summary, our study demonstrates that the bacterial methyltransferase SssI can be used to target CG methylation in plants and has revealed the chromatin features favorable for the efficient gain of methylation. This tool targets highly heritable methylation and could be used to generate epialleles of agronomical interest. The combination of this bacterial enzyme with more specific tools, like CRISPR or TAL, should improve specificity when targeting methylation for future applied uses.

## Methods

### Plant materials

All plants in this study were grown under long-day conditions (16 h light/8 h dark). The *fwa-4* mutant has been described previously^33^, as have the *fwa nrpd1*, *fwa drm1/2*, and *fwa nrpe1* lines^34^. The pMDC123-ZF-3xFLAG-SssI plasmid was transformed into Agrobacterium AGLO and then into the different backgrounds by Agrobacterium-mediated floral dipping. T1 transgenic plants were grown on 1/2 MS medium + Glufosinate 50 μg/mL (Goldbio) in growth chambers under long-day conditions and transplanted to soil. The selection of transgenic lines for experiments was based on (i) early flowering T1 plants for the lines in the *fwa* backgrounds and (ii) protein expression by Western Blot for the lines in the Col-0 background. Following transgenic generations were germinated directly on soil and the presence of the transgene was ascertained by genotyping. Plants were not selected for homozygosity except for the T4 (+) populations used for the ChIP experiment. Controls in this study correspond to untransformed plants of different backgrounds used coming from the same seed stock. Flowering time was scored by counting the total number of rosette and cauline leaves. In the flowering time dot plots, each dot represents the flowering time of individual plants. Plants with 20 or fewer leaves were considered early flowering. The samples used for all of our genomics data correspond to inflorescence tissue collected during the day.

### Plasmid construction

A plant codon-optimized cDNA sequence of the CG-specific methyltransferase gene from *Spiroplasma* sp. strain MQ1 (M.SssI) was generated and ordered from Integrated DNA Technologies (IDT) and cloned into the pENTR/D plasmid (Invitrogen) to generate pENTR/D-SssI. This plasmid was used to deliver SssI into pMDC123-UBQ10-3xFLAG-ZF^38^, a modified pMDC123 plasmid^63^ containing the *Arabidopsis* UBQ10 promoter followed by a BLRP-ZF-3xFLAG cassette located upstream of a gateway cassette, to create pMDC123-ZF-3xFLAG-SssI. The ZF contains six zinc fingers that recognize an 18 bp sequence found in each of the two small tandem repeats (CGGAAAGATGTATGGGCT) in the *FWA* promoter as described before^33^.

### BS-PCR-seq

BS-PCR-seq was performed as previously described^34^. Briefly, leaf tissues from adult plants of representative T2 lines containing the ZF-SssI transgene were collected and DNA was extracted following a CTAB-based method. Bisulfite conversion was done using the Epitect Bisulfite Conversion kit (QIAGEN). The following regions of *FWA* were analyzed: Region 1 (chr4: 13038143-13038272), Region 2 (chr4: 13038356-13038499), and Region3 (chr4: 13038568-13038695); which cover fragments of the promoter and 5′ transcribed region of *FWA*. Pfu Turbo Cx (Agilent) was used to amplify bisulfite-treated DNA using primers containing Illumina adaptors. The primers used are listed in Supplementary Data 10.

For each sample, individual PCR products from each of the three *FWA* regions were pooled and purified using AMPure beads (Beckman Coulter) before making the libraries. Libraries were made from purified PCR products using a TruSeq Nano DNA Library Prep kit for Neoprep automated library preparation machine (Illumina), a Kapa DNA hyper kit (Kapa Biosystems) with Illumina TruSeq DNA adapters, or an Ovation Ultralow V2 kit (NuGEN).

### qRT-PCR

Two-week-old *Arabidopsis* seedlings were collected for RNA extraction following the manufacturer’s instructions for the Direct-zol RNA Microprep kit (ZYMO Research). For each sample, 1 μg of RNA was converted into cDNA using SuperScript IV Reverse Transcriptase (Invitrogen) that was used as a template to perform real-time PCR using SYBR Green Master Mixes (Bio-Rad) and CFX Connect Real-Time PCR Detection System (Bio-Rad). The primers are provided in Supplementary Data 10.

### RNA-seq

Total RNA from inflorescences was extracted using the Direct-zol kit (ZYMO research). To prepare the libraries, 1 μg of total RNA was used as input for the TruSeq Stranded mRNA kit (Illumina). We performed RNA-seq in four biological replicates of Col-0 as well as two independent ZF-SssI transgenic lines in both T2 and T3 generations, either with + or without − the transgene (for T2 ZF-SssI (+) line 1, only three biological replicates were collected; *n* = 35 in total, Supplementary Data 5).

### WGBS

For T2 and T3 WGBS, DNA from inflorescences of adult plants was extracted following a CTAB-based method. Hundred nanograms of DNA were sheared to 200 bp with a Covaris S2 (Covaris) and used for library preparation using the Epitect Bisulfite Conversion kit (QIAGEN) and the Ovation Ultralow Methyl-seq kit (NuGEN) following the manufacturer’s instructions. For T4 and T5 WGBS, DNA from inflorescences of adult plants was extracted using the DNeasy Plant Mini Kit (QIAGEN). Two hundred fifty nanograms of DNA were sheared to 200 bp with a Covaris S2 (Covaris) and used for library preparation using the Epitect Bisulfite Conversion kit (QIAGEN) and the Kapa DNA hyper kit (Kapa Biosystems) with Illumina TruSeq DNA adapters following the manufacturer’s instructions.

### ChIP-seq

ChIPs were performed as described previously^34^. Briefly, 2 grams of inflorescences from untransformed Col-0 and T4 homozygous populations of two independent transgenic lines expressing ZF-SssI were ground in liquid nitrogen and fixed for 10 min in Nuclei Isolation buffer containing 1% formaldehyde. After stopping the reaction with glycine, nuclei were isolated, chromatin was sheared using a Bioruptor Plus (Diagenode), and immunoprecipitated overnight at 4 °C with the following antibodies: Anti-FLAG M2 (5 ul/ChIP used, F1804, Sigma), H3K4me1 (20 ul/ChIP used,Ab8895, Abcam), H3K4me3 (5 ul/ChIP used, 04-745, Millipore), H3K36me3 (10 ul/ChIP used, Ab9050, Abcam), H3K27me3 (10 ul/ChIP used, 07-449, Millipore), H3 (5 ul/ChIP used, Ab1791, Abcam), H2A (10 ul/ChIP used, Ab13923, Abcam), H2A.Z (3 ul/ChIP used)^64,65^, and PanH3Ac (5 ul/ChIP used, 39140, Active motif). Chromatin-bound proteins were immunoprecipitated with a 1:1 mixture of magnetic Protein A and Protein G Dynabeads (Invitrogen) for 3 h at 4 °C, washed with low salt, high salt, LiCl, and TE buffers for 10 min each at 4 °C and eluted for 2 × 20 min at 65 °C with elution buffer. Reversal of crosslinks was done overnight at 65 °C, followed by proteinase K treatment at 45 °C for 5 h. DNA was purified using Phenol:Chloroform:Isoamyl Alcohol 25:24:1 (Fisher Scientific) and precipitated with NaAc/EtOH and GlycoBlue (Invitrogen) overnight at −20 °C. Libraries were prepared using the Ovation Ultra Low System V2 1-16 kit (NuGEN) following the manufacturer’s instructions.

### ATAC-seq

ATAC-seq libraries were prepared as previously described^64,66^. Briefly, inflorescence tissues were first collected for nuclei extraction^16,67^. Then a transposition reaction was conducted in 25 µL of 2× DMF buffer (66 mM Tris-acetate (pH = 7.8), 132 K-Acetate, 20 mM Mg-Acetate, and 32% DMF) mixed with 2.5 µL of Tn5 and 22.5 µL of nuclei suspension and incubated at 37 °C for 30 min. Transposed DNA fragments were then purified with ChIP DNA Clean & Concentrator Kit (Zymo, cat. no. D5205). Libraries were then amplified as described before^64,66,68^.

### BS-PCR-seq analysis

BS-PCR-seq analysis was conducted as previously described^34^. Briefly, raw sequencing reads with designed BS-PCR primers were first filtered and trimmed based on the primer sequence with customized scripts. Trimmed reads were then mapped to the reference TAIR10 genome with BSMAP^69^ (v.2.74) by allowing up to two mismatches (-v 2), one best hit (-w 1), and to both strands (-n 1). The methylation level at each cytosine was then calculated with BSMAP (methratio.py) script by only keeping unique mapped reads (-u). Reads with more than three consecutive methylated CHH sites were removed^34^. Methylation levels at each cytosine were calculated as #C/(#C + #T). Cytosines with less than 20 reads coverage were excluded from further analysis. To visualize the BS-PCR-seq data, only cytosines within amplified regions were kept and plotted with R (ggplot2 package, https://ggplot2.tidyverse.org/).

### WGBS analysis

WGBS analysis was performed as previously described^34^. Briefly, raw reads were first aligned to the reference TAIR10 genome using BSMAP^69^ (v2.74) by allowing up to two mismatches (-v 2), one best hit (-w 1), and to both strands (-n 1). The methylation level at each cytosine was then calculated with BSMAP (methratio.py) script by only keeping unique mapped reads (-u). Reads with more than three consecutive methylated CHH sites were removed^49^. Methylation levels at each cytosine were calculated as #C/(#C + #T). DMRs between ZF-SssI and Col-0 were defined using the R package DMRcaller^34^. To increase coverage for DMR analysis, biological replicates were merged for each genotype (ZF-SssI (+) and ZF-SssI (‒)), each generation (T2 to T5), and each transgenic lines (line 1 and line 2). In general, the whole TAIR10 genome was divided into 200 bp bins and only bins with at least four cytosines, where each cytosine is covered at least four times, has more than 10% more methylation in ZF-SssI than Col-0, and has a significance level of less than 0.05 were kept. To define hCG DMRs for T2 and T3, the intersecting hCG DMRs of two transgenic lines in each generation were first calculated. Then the union set of T2 and T3 in the same genotype (either ZF-SssI (+) or ZF-SssI (‒)) were kept. DMRs overlapping with 200 bp bins in each cluster was considered as DMRs specific for certain clusters. Genomic locations for DMRs and mCG-equivalent control were annotated using the Homer^70^ ‘annotatePeaks’ function with default parameters. For T4 and T5, two transgenic lines were separated in order to trace the heritable hCG DMRs. To define heritable hCG DMRs, T2 ZF-SssI – were compared with T2 ZF-SssI (+) and the shared hCG DMRs were considered as heritable hCG DMRs in T2 ZF-SssI (‒). For T3 ZF-SssI (+) and ZF-SssI (‒), hCG DMRs were overlapped with T2 ZF-SssI (+). For T4 ZF-SssI (‒) heritable hCG sites, DMRs were first intersected with T3 ZF-SssI (+) and then intersected with T2 ZF-SssI (+), while T5 ZF-SssI (‒) heritable hCG sites were further intersected with T4 ZF-SssI (‒) hCG DMRs. WGBS data for controls (Col-0 and *fwa*) used for ZF-SssI in *fwa* analysis were published before in GSM2932284^38^ and GSM3553008^34^.

### ChIP-seq analysis

For ChIP-seq data, raw reads were first mapped to the reference TAIR10 genome with Bowtie^71^ (v0.12.8) by allowing uniquely mapped reads and a maximum of two mismatches. PCR-duplicated reads were then filtered with SAMTools^72^ (v 1.19) (Supplementary Data 3). To call ZF-SssI FLAG peaks, the MACS2 calldiff function^73^ (v 2.1.2) was used to compare ZF-SssI FLAG ChIP-seq and Col-0 FLAG ChIP-seq data with default parameters. Genomic location and enriched motifs of ZF-SssI FLAG-specific peaks were then annotated with Homer^70^ ‘annotatePeaks’ and ‘findMotifGenome’ functions using 100 bp flanking the summit of the peaks. Promoter regions were defined as default in homer (upstream 1 kb and downstream 100 bp of TSS). ChIP-seq peaks for histone marks in Col-0 were defined using MACS2^73^ with –nomodel and –call-summits as parameters. ChIP-seq data visualizations were performed using ngs.plot^74^, deepTools^75^, or EnrichedHeatmap^76^.

### RNA-seq analysis

For RNA-seq analysis, FastQC was first used to quality-assure the raw reads (v0.11.8). Raw reads were then aligned to the TAIR10 reference genome and TAIR10 gene annotation using STAR^77^ (v2.7.0e) with ‘–outFilterMultimapNmax 1000 –outSAMmultNmax 1’ options. Read counts over each gene were then calculated by featureCounts^78^ (v2.0.0) with default parameters. Expression levels were determined by RPKM (reads per kilobase of exons per million aligned reads) in R by customized script. Differentially expressed genes were determined with R package DESeq2^79^ using a 2-fold change and a false discovery rate (FDR) of less than 0.05 as cutoff. Alternative splicing events were analyzed using rMATS^45^ (v4.0.2) with the default parameters. Analysis for DEG associated with hCG DMR in ZF-SssI was performed using the web tool RAD^44^ (http://labw.org/rad) with default parameters.

### ATAC-seq analysis

ATAC-seq analysis was performed as previously described^64,66,68^. Briefly, paired-end reads were aligned to the TAIR10 reference genome with bowtie^71^ (v0.12.8) by allowing maximal two mismatches, uniquely mapped reads (-m 1), and the maximal 2 kb distance between pairs (-X 2000). PCR-duplicated reads were removed using SAMTools^72^ (v1.19) ‘rmdup’ function and visualized with ngs.plot^74^ or deepTools^75^.

### Reporting summary

Further information on research design is available in the Nature Research Reporting Summary linked to this article.

## Supplementary information

Supplementary Information

Supplementary Data 1

Supplementary Data 2

Supplementary Data 3

Supplementary Data 4

Supplementary Data 5

Supplementary Data 6

Supplementary Data 7

Supplementary Data 8

Supplementary Data 9

Supplementary Data 10

Description of Additional Supplementary Files

Reporting Summary

## Data Availability

Data supporting the findings of this work are available within the paper and its Supplementary Information files. A reporting summary for this Article is available as a Supplementary Information file. The datasets and plant materials generated and analyzed during the current study are available from the corresponding author upon request. All high-throughput sequencing data generated is accessible at NCBI’s Gene Expression Omnibus (GEO) via GEO Series accession number GSE158027. WGBS data for controls (Col-0 and *fwa*) used for ZF-SssI in *fwa* analysis were previously published under accession GSM2932284^38^ and GSM3553008^34^. Source data are provided with this paper.

## Source data

Source Data

## Code availability

Customized code/scripts used for BS-PCR-seq analysis have been deposited on GitHub [https://github.com/wanluliu/BSPCR-analysis-for-FWA].
