# Supplementary material for: Ectopic targeting of CG DNA methylation in Arabidopsis with the bacterial SssI methyltransferase
Source: Nat Commun. 2021 May 25;12:3130. doi: 10.1038/s41467-021-23346-y (PMC8149686; doi:10.1038/s41467-021-23346-y)
Supplement: Supplementary file 1 — Supplementary Information [file 41467_2021_23346_MOESM1_ESM.pdf]

Ectopic targeting of CG DNA methylation in Arabidopsis with the bacterial SssI methyltransferase

Liu and Gallego-Bartolomé *et al.*

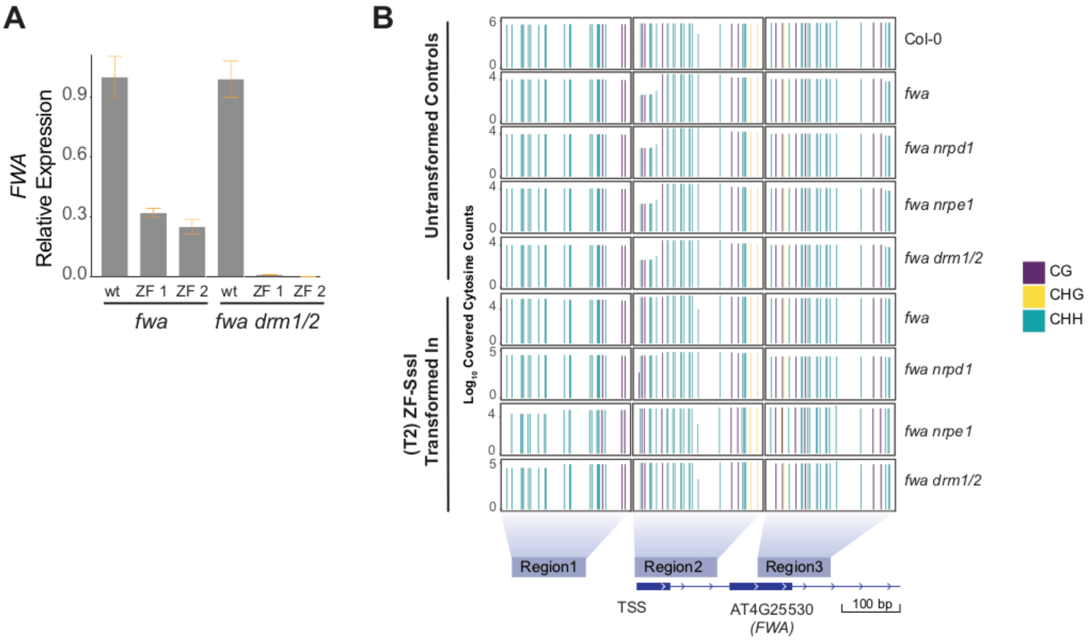

Supplementary Figure 1. SssI targeted methylation cause FWA silencing.
A. Relative FWA gene expression measured by qRT-PCR in 10 day-old T2 seedlings from *fwa*, *fwa drm1/2*, and two representative ZF-SssI lines in the *fwa* background and *fwa drm1/2* background. Three technical replicates were performed for each qRT-PCR reaction. Error bar represents standard deviations. Data are presented as mean values ± SD. **B.** BS-PCR-seq read coverages for each cytosine in samples from untransformed controls and ZF-SssI plants in the *fwa* background or mutants that have been introgressed into the *fwa* background. The bar plot represents data from one representative T2 plant for each genotype tested. Every single bar represents one cytosine. The relative position of the three regions analyzed in the *FWA* gene are indicated as blue squares. Source data underlying Supplementary Figure 1A are provided as a Source Data file.

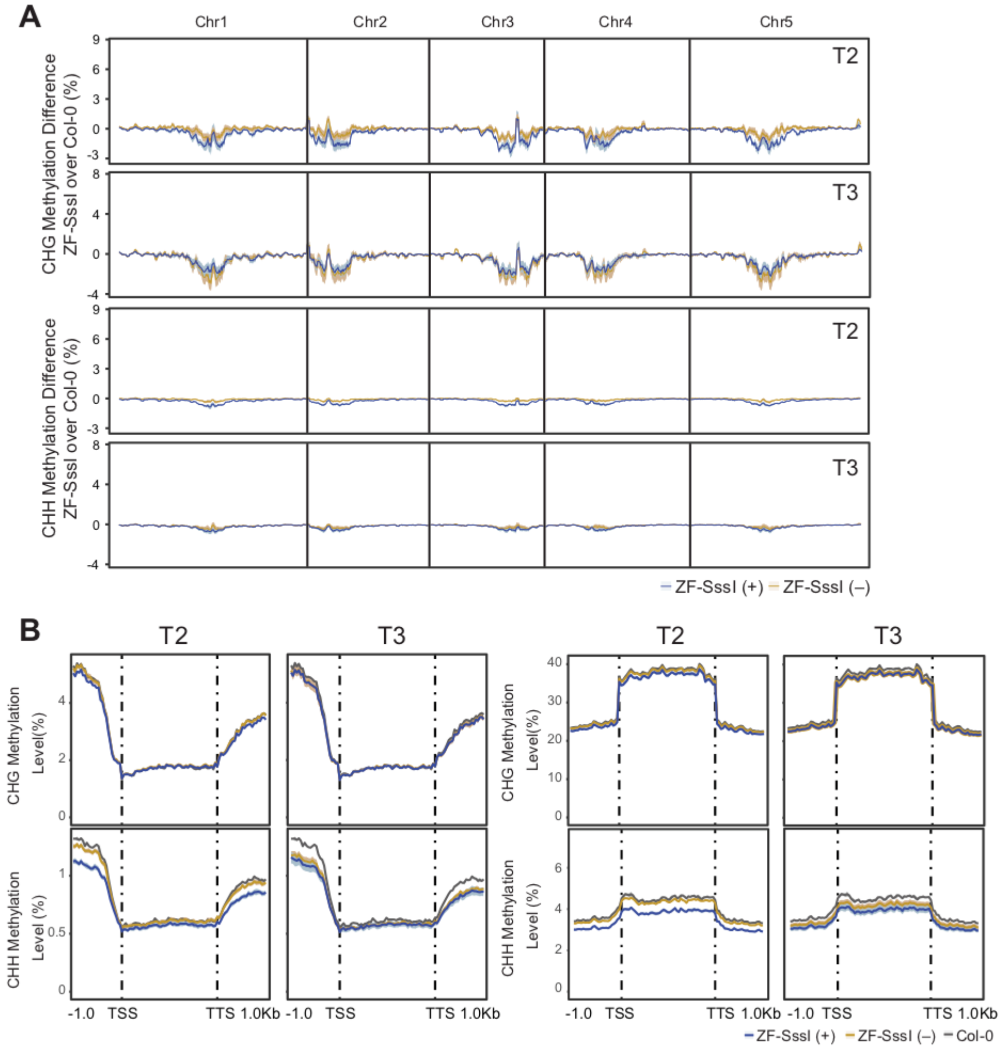

Supplementary Figure 2. No CHG or CHH methylation is targeted by SssI.
A. Genome-wide CHG and CHH methylation difference in ZF-SssI lines during T2 and T3 with (+) or without (–) the transgene. The curve represents the mean, shaded area around the curve represents standard errors (n=4). **B.** Metaplot of CHG and CHH methylation over protein-coding genes (left two panels) or transposable elements (TE) (right two panels) in Col-0 and ZF-SssI lines during T2 and T3 with (+) or without (–) the transgene. The curve represents the mean, shaded area around the curve represents standard errors (n=4).

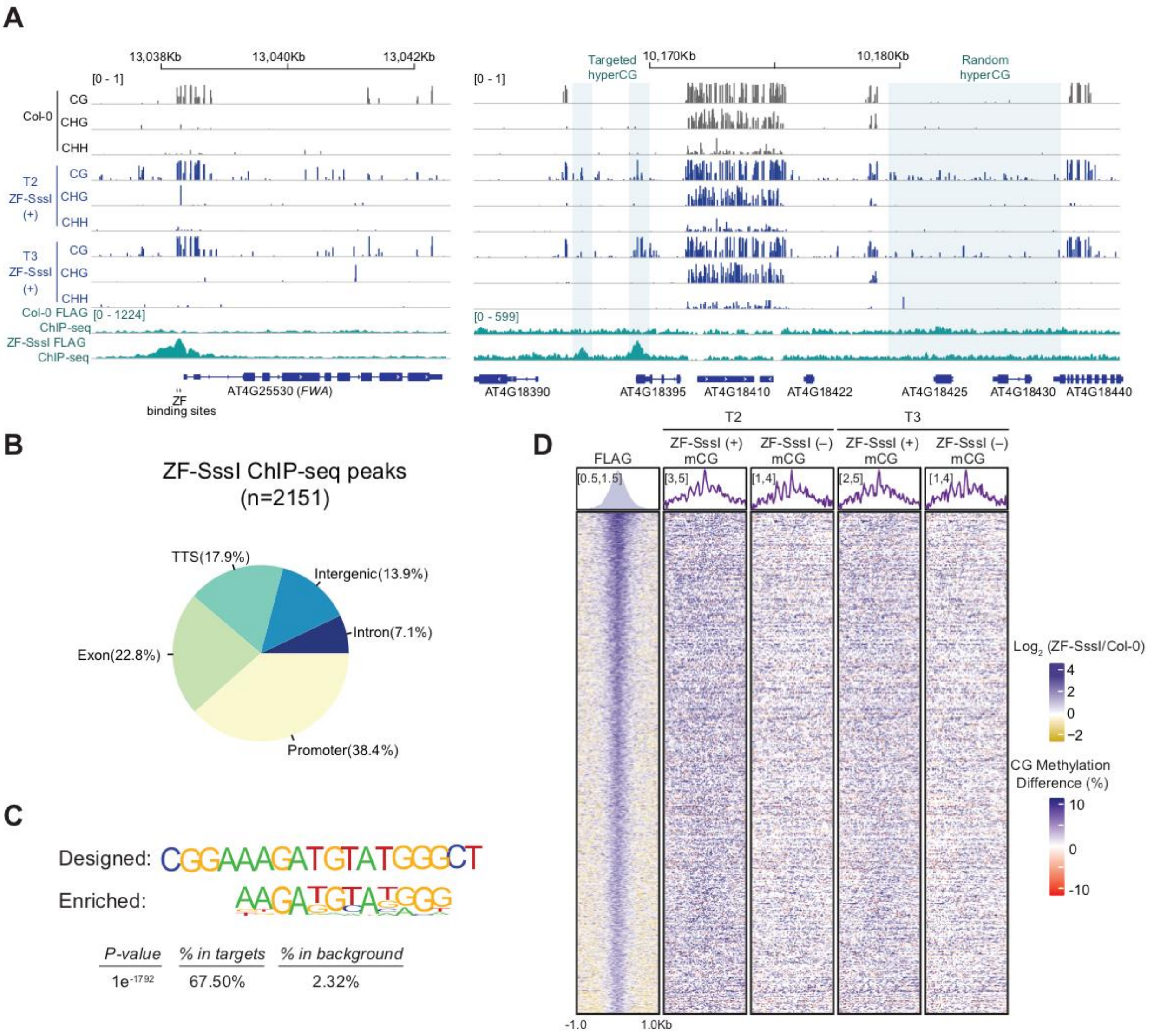

Supplementary Figure 3. SssI is directed to thousands of loci.
A. Screenshot of WGBS and ChIP-seq signals in Col-0 and representative ZF-SssI lines during T2 and T3 with the transgene over *FWA* (left) and a selected genomic region (right) showing ZF-SssI-bound hyperCG (targeted hyperCG) as well as ZF-SssI-not-bound hyperCG (random hyperCG). Black triangles indicate designed ZF binding sites. **B.** Pie chart of the genomic distribution of ZF-SssI ChIP-seq peaks. **C.** *De novo* motif analysis over ZF-SssI ChIP-seq peaks by Homer. *P-value* calculated by Homer with default parameters. **D.** Heatmap and metaplot of ZF-SssI ChIP-seq, and T2 and T3 CG methylation difference compared to Col-0 in ZF-SssI with (+) or without (–) the transgene over ZF-SssI ChIP-seq peaks. Numbers within the square brackets represent the scales of the metaplot.

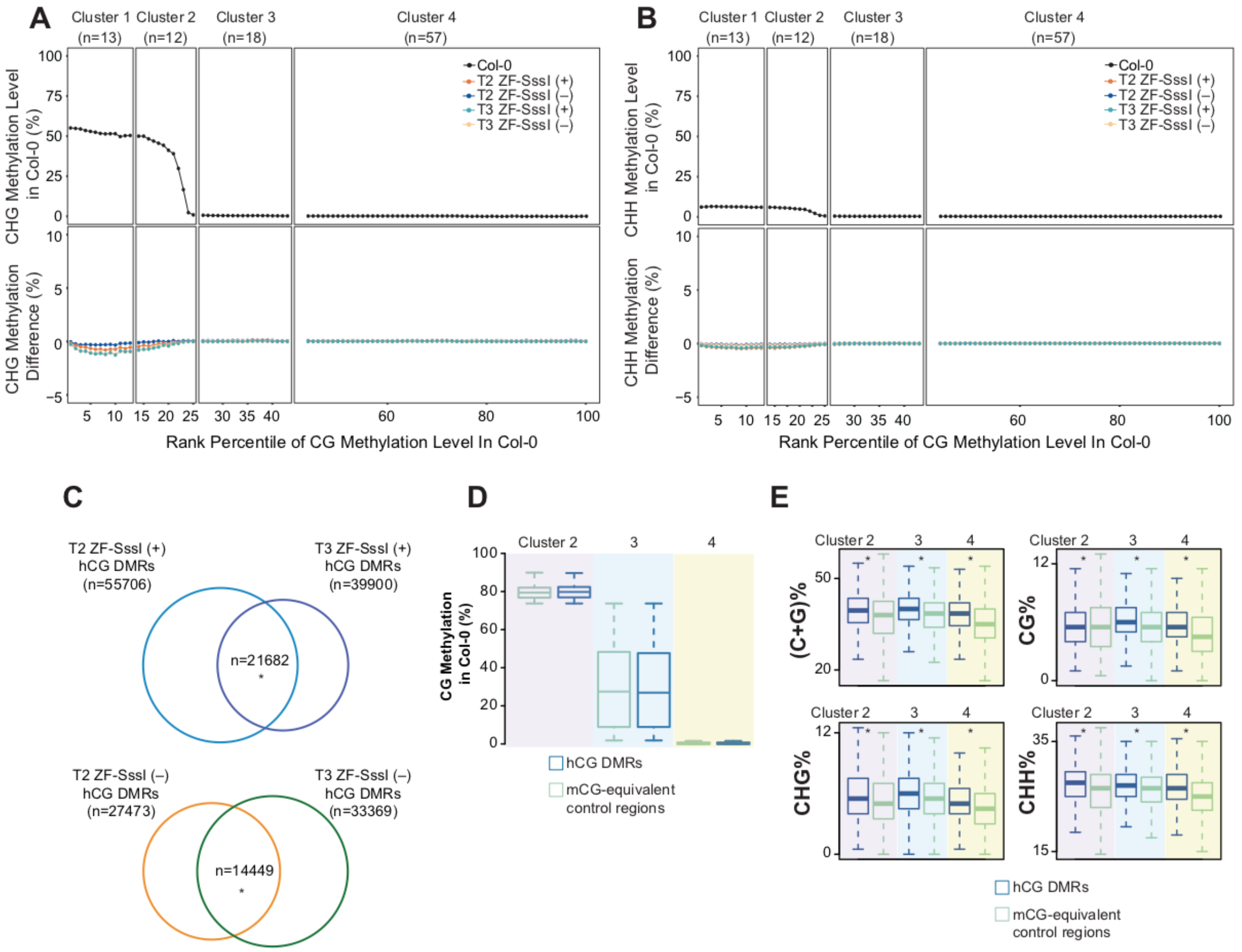

Supplementary Figure 4. Hyper CG DMRs are largely shared between T2 and T3.
A, B. CHG (A) and CHH (B) methylation level of 200 bp bins in Col-0 (upper panels), CHG and CHH methylation difference in ZF-SssI lines during T2 and T3 with (+) or without (–) the transgene (lower panels) ranked by the mCG level in Col-0. Methylation difference is the absolute difference level between ZF-SssI lines and Col-0. Four clusters are consistent with Figure 3B, defined by the methylation level of 200 bp bins in Col-0. **C.** The number of overlapped hCG DMRs combining Clusters 2, 3, and 4 in ZF-SssI lines during T2 and T3 with (+) or without (–) the transgene. (* *p-value* < 2.26×10^{-16}, one-sided hypergeometric test). **D.** mCG level in Col-0 for hCG DMR and mCG-equivalent control regions in Clusters 2, 3, and 4. **E.** Boxplot of (C+G)%, CG, CHG, and CHH density (CG%, CHG%, and CHH%) over hCG DMRs and mCG-equivalent control regions in Clusters 2, 3, and 4. To control for CG methylation in Col-0, a random control is selected from 200 bp bins within the same percentile in Figure 3B (* *p-value* <0.01, Welch two-sample t-test). For boxplots in D and E, the middle line shows the median; boxes represent the 25th (bottom) and 75th (top) percentiles; and bars represent the minimum and maximum points within the 1.5X interquartile range. For D and E, Clusters 2, 3, and 4 hCG DMR counts are 2044, 22631, and 48044. Source data underlying Supplementary Figure 4C and 4E are provided as a Source Data file.

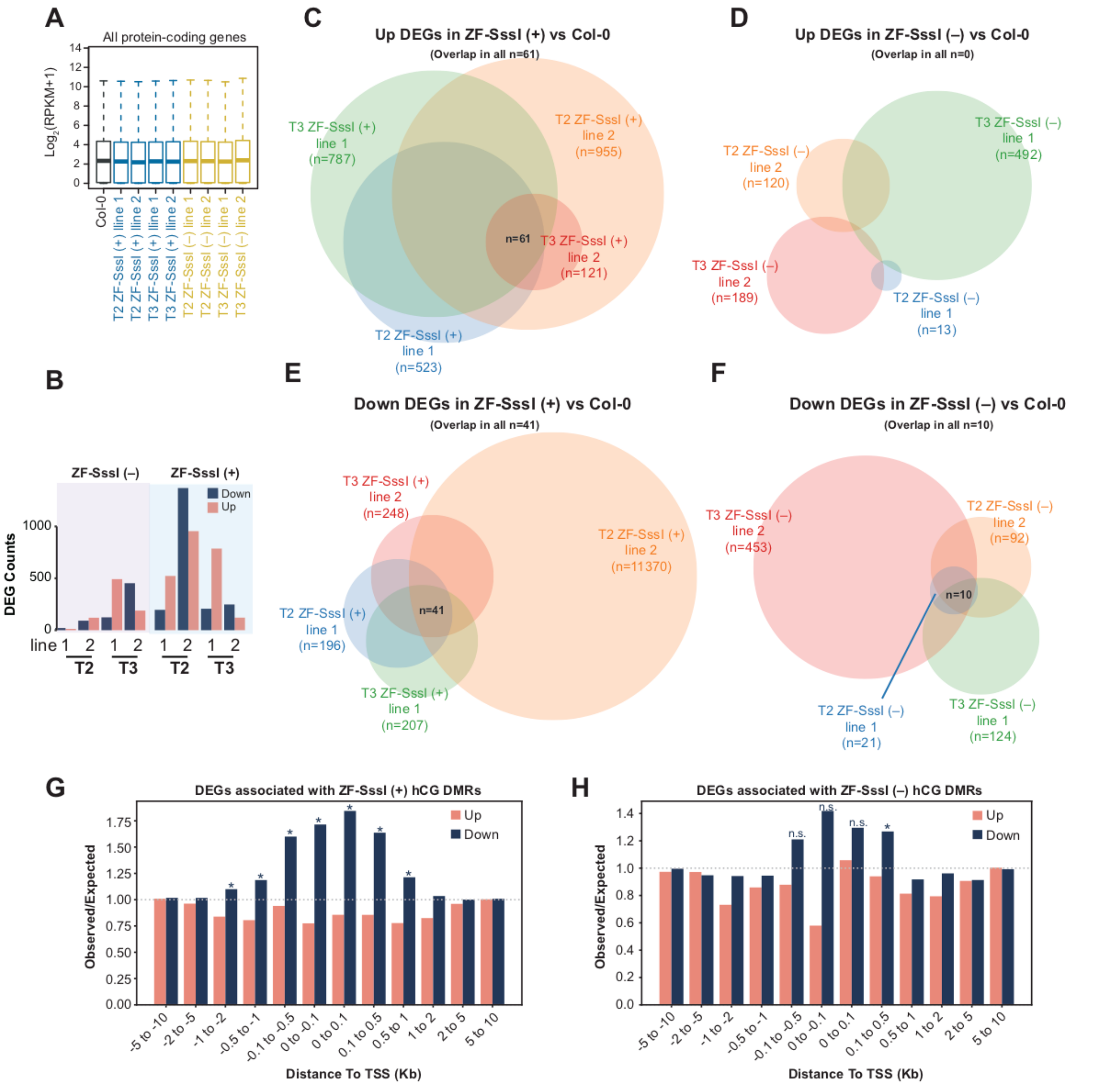

Supplementary Figure 5. hCG triggers limited changes in gene expression.
A. Boxplot of the expression level for all genes in Col-0 and ZF-SssI lines during T2, and T3 with (+) or without (–) the transgene. The middle line shows the median; boxes represent the 25th (bottom) and 75th (top) percentiles; and bars represent the minimum and maximum points within 1.5X interquartile range. n=4 for Col-0 as well as two independent ZF-SssI transgenic lines in both T2 and T3 generations, either with + or without - the transgene (for T2 ZF-SssI (+) line 1, only three biological replicates were collected; n=35 in total). **B.** Count of DEGs in ZF-SssI lines with (+) or without (–) the transgene compared with Col-0. **C-F.** Venn diagrams of up- and down-regulated DEGs in ZF-SssI lines during T2 and T3 with (+) or without (–) the transgene compared with Col-0. **G, H.** Bar plot representing the association of hCG DMRs and DEGs in ZF-SssI lines with (+) or without (–) the transgenes analyzed by RAD (Region Associated DEG, https://labw.org/rad) (* *p-value* < 0.05, one-sided hypergeometric test; n.s. means not significant).

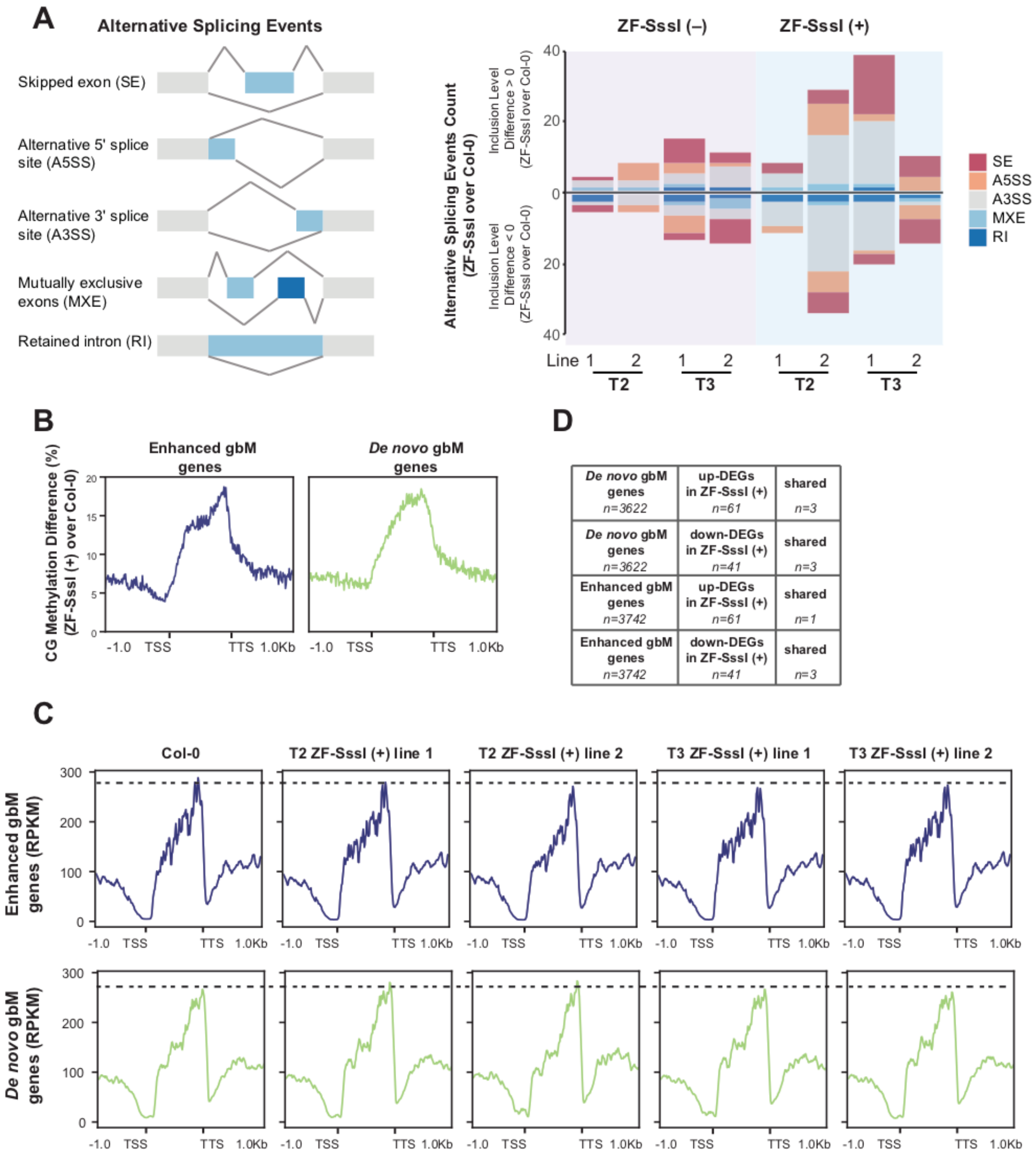

Supplementary Figure 6. Limited transcriptional changes over 'Enhanced gbM' or '*De novo* gbM' genes.

A. Alternative splicing events analyzed with rMATs in ZF-SssI lines during T2 and T3 with (+) or without (–) the transgene compared with Col-0. **B.** Metaplot of the CG methylation difference between ZF-SssI lines with the transgene (+) and Col-0 over protein-coding genes with 'Enhanced gbM' or '*De novo* gbM'. **C.** Metaplot of gene expression levels for RNA-seq data in Col-0 and ZF-SssI (+) lines during T2 and T3 over protein-coding genes with 'Enhanced gbM' or '*De novo* gbM'. **D.** Counts of 'Enhanced gbM', '*De novo* gbM', up- or down-regulated DEGs in ZF-SssI (+) compared to Col-0, and the overlap between DEGs and genes with hCG. Source data underlying Supplementary Figure 6A are provided as a Source Data file.

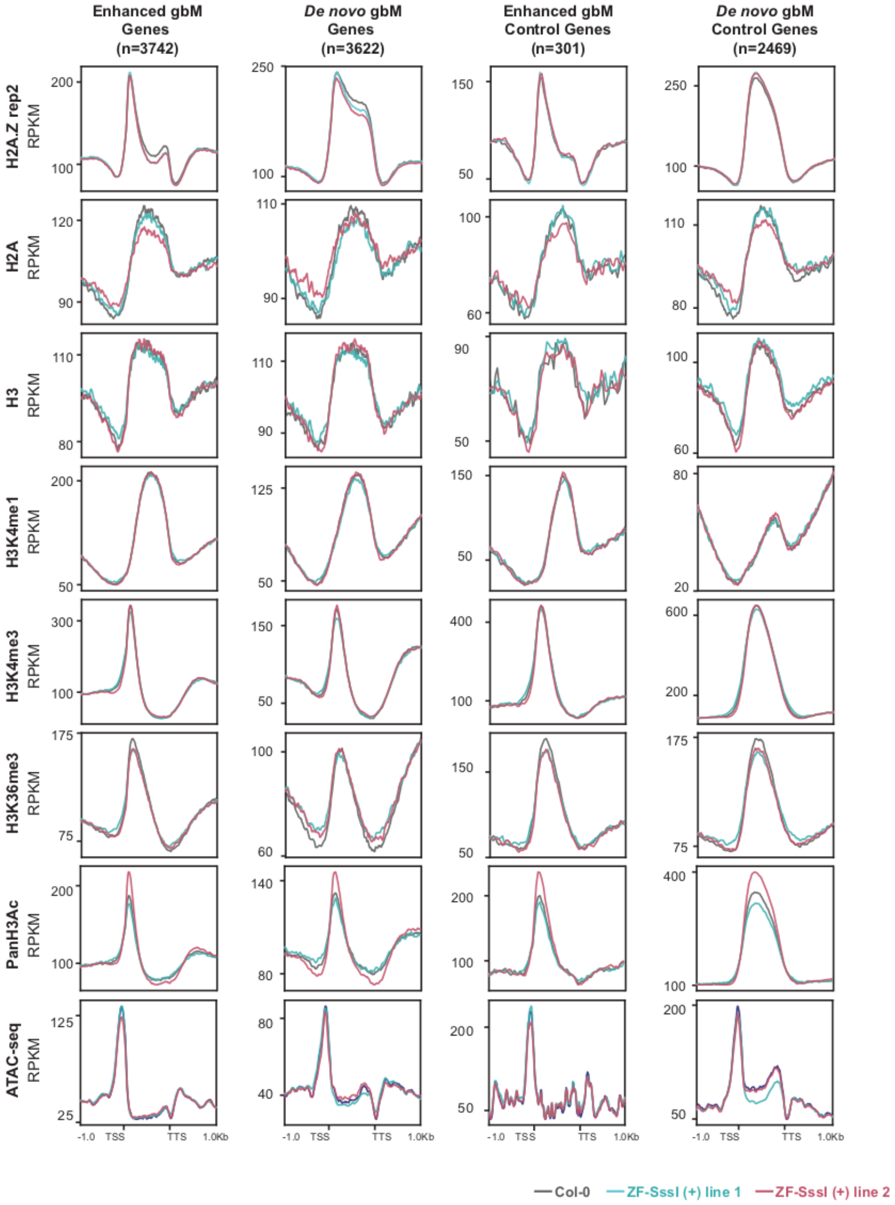

Supplementary Figure7. Selected histone marks and chromatin accessibility over ‘Enhanced gbM’ or ‘*De novo* gbM’ genes.

Metaplot of H2A.Z (replicate 2), H2A, H3, H3K4me1, H3K4me3, H3K36me3, PanH3Ac signals, and ATAC-seq signals in Col-0 and two ZF-SssI (+) lines over ‘Enhanced gbM’, ‘*De novo* gbM’ genes and their control.

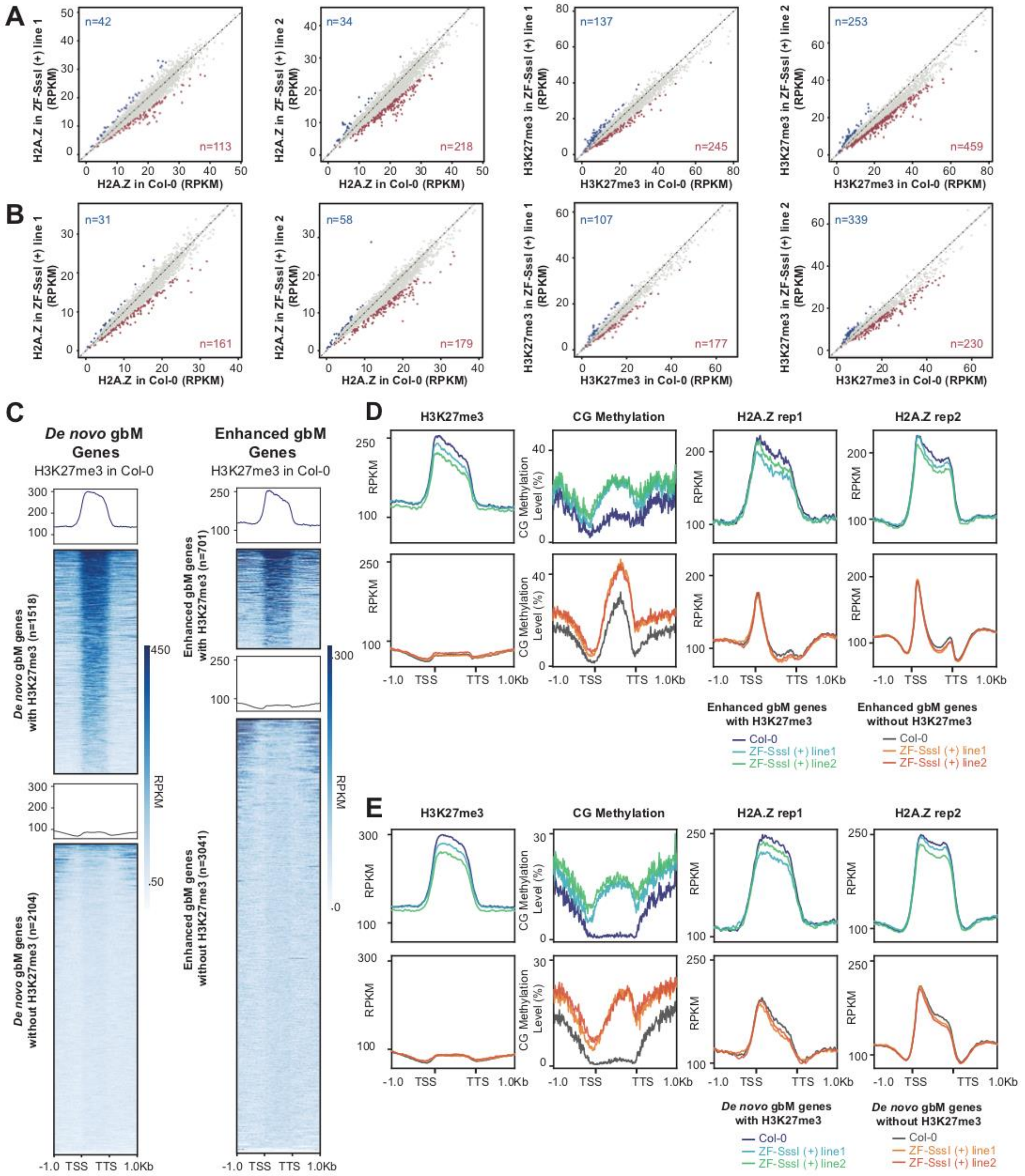

Supplementary Figure 8. H2A.Z, mCG methylation, and H3K27me3 distribution over '*De novo* gbM' or 'Enhanced gbM' genes with or without H3K27me3.

A-B. Scatterplot of H2A.Z and H3K27me3 signals in Col-0 and ZF-SssI (+) lines over '*De novo* gbM' genes (A) or 'Enhanced gbM' genes (B). Colored dots represent genes with reduced (red dots, fold change ≤ 0.8), increased (blue dots, fold change ≥ 1.25), or no change (grey dots) for H2A.Z or H3K27me3 signals in ZF-SssI (+) lines compared to Col-0. **C.** Metaplot and heatmap of H3K27me3 in Col-0 for '*De novo* gbM' genes and 'Enhanced gbM' genes classified as with or without H3K27me3. **D.** Metaplot of H3K27me3, CG methylation level, and H2A.Z level in Col-0 and two representative ZF-SssI (+) lines over 'Enhanced gbM' genes with or without H3K27me3 in Col-0. **E.** Metaplot of H3K27me3, CG methylation level, and H2A.Z level in Col-0 and two representative ZF-SssI (+) lines over '*De novo* gbM' genes with or without H3K27me3 in Col-0.

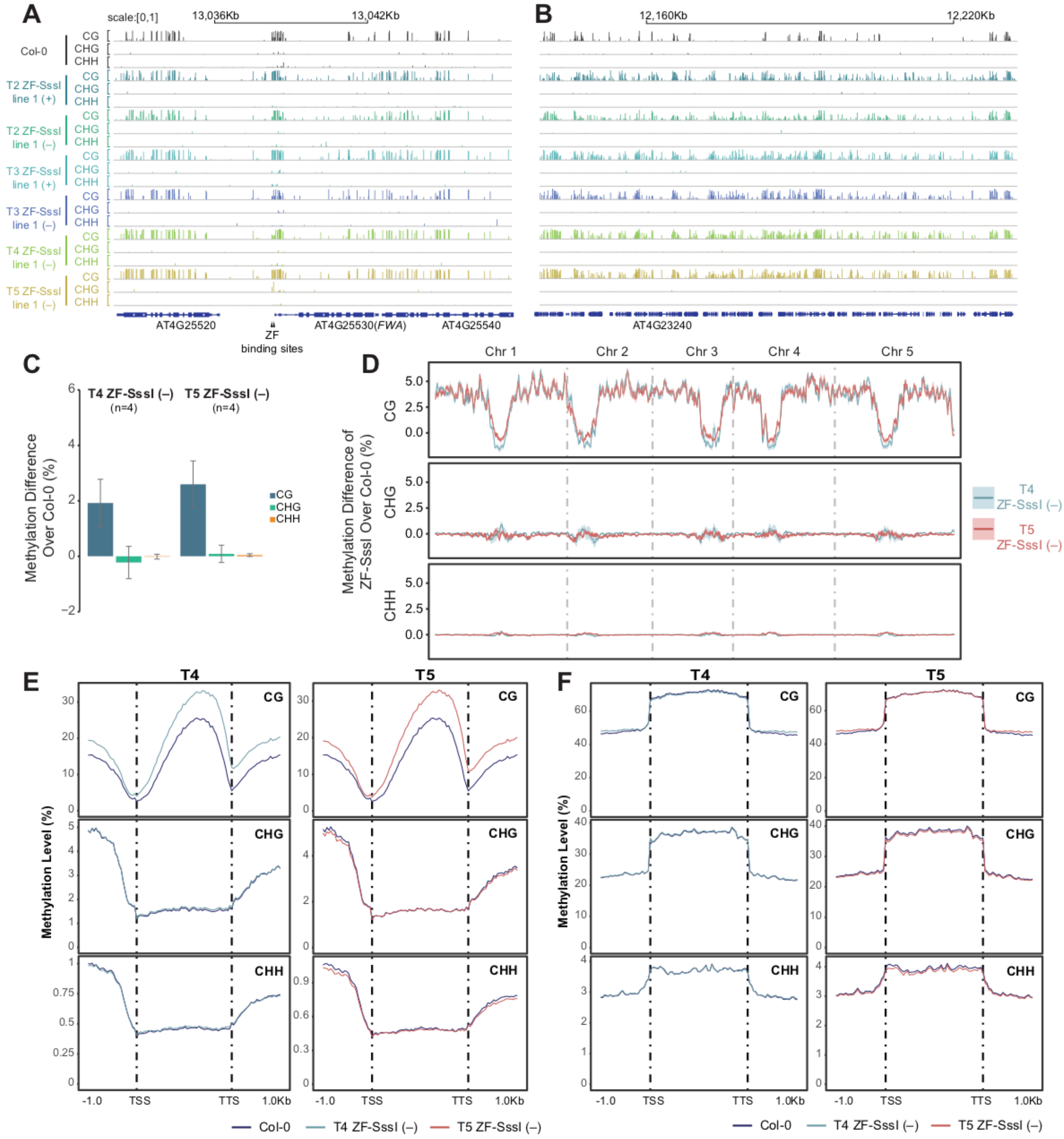

Supplementary Figure 9. Targeted CG methylation is heritable over multiple generations. A, B. Screenshot of CG, CHG, and CHH methylation in representative ZF-SssI line 1 during T2 to T5 with (+) or without (–) the transgene in Col-0 background over *FWA* (A) and a selected genomic region (B). Every bar represents a single base pair. The black triangles indicate designed ZF binding sites. **C.** Bar plot of genome-wide CG, CHG, and CHH methylation difference for T4 and T5 ZF-SssI without (–) the transgene over the corresponding Col-0. Error bars represent standard errors, center of error bars represents mean. **D.** Genome-wide metaplot of CG, CHG, and CHH methylation difference for ZF-SssI without (–) the transgene during T4 and T5 over the corresponding Col-0. The curve represents the mean, shaded area around the curve represents standard errors (n=4). **E, F.** CG, CHG, and CHH methylation metaplot for ZF-SssI without the transgene (–) during T4 or T5 and Col-0 over protein-coding genes (E) or transposable elements (F).

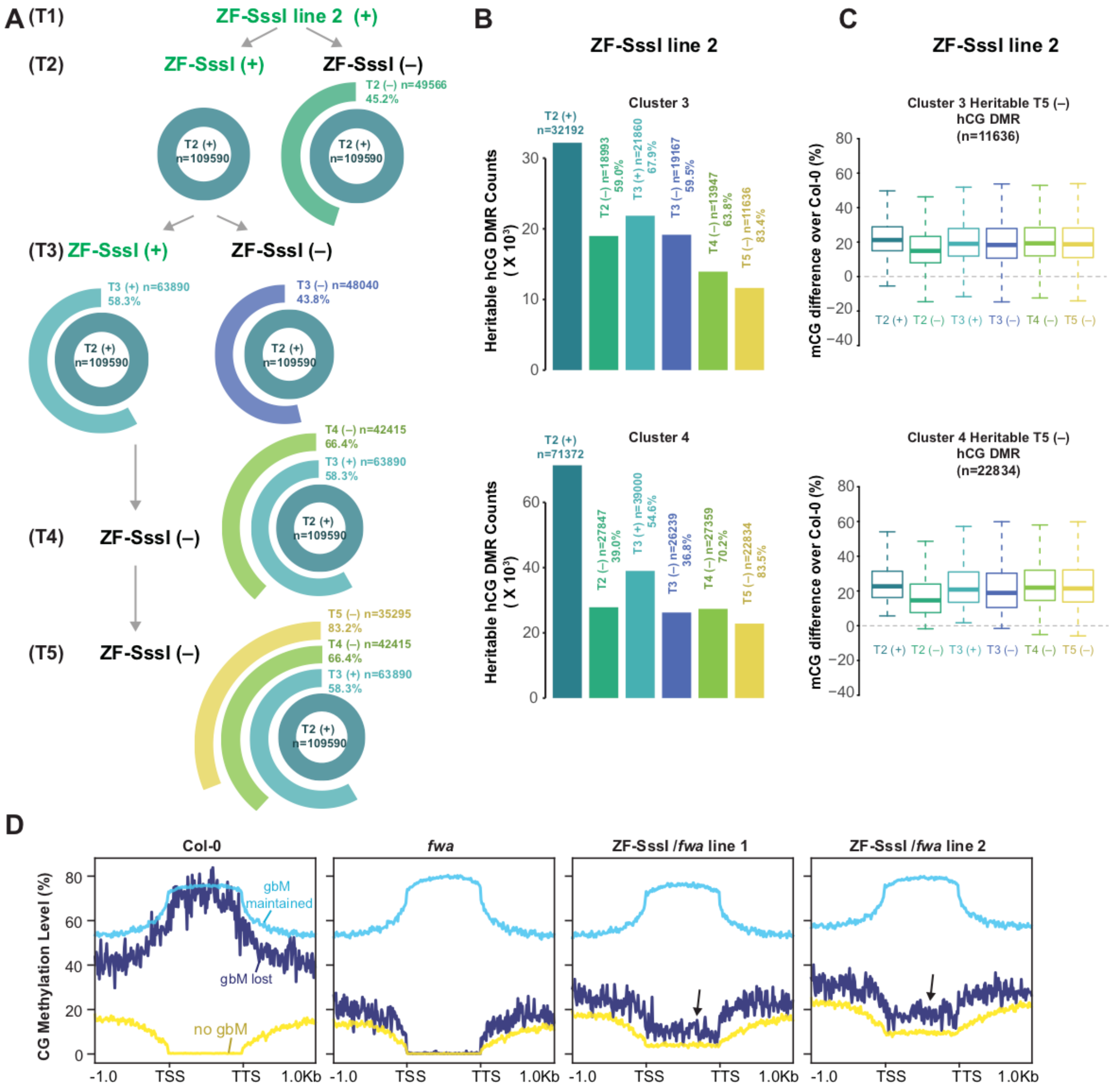

Supplementary Figure 10. Targeted CG methylation is heritable in ZF-SssI line 2.
A. Multilevel pie chart of the number of heritable hCG DMRs in ZF-SssI line 2 during T2, T3, T4, and T5. **B.** Bar plot for the number of heritable hCG DMRs over Clusters 3 and 4 in ZF-SssI line 2 during T2, T3, T4, and T5. For percentage of heritable hyperCG DMRs, T2 (–), T3 (+), and T3 (–) are compared with T2 (+); T4 (–) is compared with T3 (+); T5 (–) is compared with T4 (–). **C.** Boxplot of CG methylation difference level of ZF-SssI during T2 to T5 over Col-0 in Clusters 3, and 4 heritable hCG DMRs in ZF-SssI line 2 (–) during T5. The middle line shows the median; boxes represent the 25th (bottom) and 75th (top) percentiles; and bars represent the minimum and maximum points within the 1.5X interquartile range. **D.** Metaplot of CG methylation in Col-0, *fwa*, and two ZF-SssI lines in the *fwa* background over 'no gbM', 'gbM lost', and 'gbM maintained' transposable elements (TEs). 'no gbM' represents TEs with no gbM in *fwa* and Col-0; 'gbM lost' represents TEs that lost gbM in *fwa* compared with Col-0; 'gbM maintained' represents TEs that maintained gbM in *fwa* compared to Col-0. The black arrow indicates the hCG in ZF-SssI lines in the *fwa* background over TEs that had lost gbM in *fwa*.
