# Supplementary material for: Ectopic targeting of CG DNA methylation in Arabidopsis with the bacterial SssI methyltransferase
Source: Nat Commun. 2021 May 25;12:3130. doi: 10.1038/s41467-021-23346-y (PMC8149686; doi:10.1038/s41467-021-23346-y)
Supplement: Supplementary file 12 — Description of Additional Supplementary Files [file 41467_2021_23346_MOESM12_ESM.pdf]

Description of additional supplementary files

Title: Supplementary Data 1

Description: WGBS information and alignment summary.

Title: Supplementary Data 2

Description: hCG DMR list.

Title: Supplementary Data 3

Description: ChIP-seq information and alignment summary.

Title: Supplementary Data 4

Description: ATAC-seq information and alignment summary.

Title: Supplementary Data 5

Description: RNA-seq information and alignment summary.

Title: Supplementary Data 6

Description: Up- and down-regulated DEGs list in ZF-SssI + and ZF-SssI -.

Title: Supplementary Data 7

Description: RAD results for hCG DMRs and DEGs.

Title: Supplementary Data 8

Description: gbM gene list.

Title: Supplementary Data 9

Description: H2A.Z and H3K27me3 levels over 'de novo gbM' and 'enhanced gbM' genes.

Title: Supplementary Data 10

Description: Primers used in this study.
