# Supplementary material for: Ectopic targeting of CG DNA methylation in Arabidopsis with the bacterial SssI methyltransferase
Source: Nat Commun. 2021 May 25;12:3130. doi: 10.1038/s41467-021-23346-y (PMC8149686; doi:10.1038/s41467-021-23346-y)
Supplement: Supplementary file 13 — Reporting Summary [file 41467_2021_23346_MOESM13_ESM.pdf]

nature research

Corresponding author(s): Steven E. Jacobsen, Wanlu Liu

Last updated by author(s): Dec 22, 2020

Reporting Summary

Nature Research wishes to improve the reproducibility of the work that we publish. This form provides structure for consistency and transparency in reporting. For further information on Nature Research policies, see our Editorial Policies and the Editorial Policy Checklist.

Statistics

For all statistical analyses, confirm that the following items are present in the figure legend, table legend, main text, or Methods section.

n/a	Confirmed	
☐	☒	The exact sample size (*n*) for each experimental group/condition, given as a discrete number and unit of measurement
☐	☒	A statement on whether measurements were taken from distinct samples or whether the same sample was measured repeatedly
☐	☒	The statistical test(s) used AND whether they are one- or two-sided<br>*Only common tests should be described solely by name; describe more complex techniques in the Methods section.*
☒	☐	A description of all covariates tested
☐	☒	A description of any assumptions or corrections, such as tests of normality and adjustment for multiple comparisons
☐	☒	A full description of the statistical parameters including central tendency (e.g. means) or other basic estimates (e.g. regression coefficient) AND variation (e.g. standard deviation) or associated estimates of uncertainty (e.g. confidence intervals)
☐	☒	For null hypothesis testing, the test statistic (e.g. *F*, *t*, *r*) with confidence intervals, effect sizes, degrees of freedom and *P* value noted<br>*Give P values as exact values whenever suitable.*
☒	☐	For Bayesian analysis, information on the choice of priors and Markov chain Monte Carlo settings
☒	☐	For hierarchical and complex designs, identification of the appropriate level for tests and full reporting of outcomes
☒	☐	Estimates of effect sizes (e.g. Cohen's *d*, Pearson's *r*), indicating how they were calculated

Our web collection on statistics for biologists contains articles on many of the points above.

Software and code

Policy information about availability of computer code

Data collection

No software were used for data collection.

Data analysis

BS-PCR-seq analysis
BS-PCR-seq analysis was conducted as previously described. Briefly, raw sequencing reads with designed BS-PCR primers were first filtered and trimmed based on the primer sequence with customized scripts. Trimmed reads were then mapped to the reference TAIR10 genome with BSMAP 66 (v.2.74) by allowing up to two mismatches (-v 2), one best hit (-w 1), and to both strands (-n 1). The methylation level at each cytosine was then calculated with BSMAP (methratio.py) script by only keeping unique mapped reads (-u). Reads with more than three consecutive methylated CHH sites were removed as previously described. Methylation levels at each cytosine were calculated as #C/(#C+#T). Cytosines with less than 20 reads coverage were excluded from further analysis. To visualize the BS-PCR-seq data, only cytosines within amplified regions were kept and plotted with R (ggplot2 package, https://ggplot2.tidyverse.org/). The customized code used for BS-PCR-seq analysis have been deposited to GitHub (https://github.com/wanluliu/BSPCR-analysis-for-FWA).

WGBS analysis
WGBS analysis was performed as previously described. Raw reads were first aligned to the reference TAIR10 genome using BSMAP (v2.74) by allowing up to two mismatches (-v 2), one best hit (-w 1), and to both strands (-n 1). The methylation level at each cytosine was then calculated with BSMAP (methratio.py) script by only keeping unique mapped reads (-u). Reads with more than three consecutive methylated CHH sites were removed as previously described . Methylation levels at each cytosine were calculated as #C/(#C+#T). DMRs between ZF-SssI and Col-0 were defined as previously described using the R package DMRcaller. To increase coverage for DMR analysis, biological replicates were merged for each genotype (ZF-SssI + and ZF-SssI -), each generation (T2 to T5), and each transgenic lines (line 1 and line 2). In general, the whole TAIR10 genome was divided into 200 bp bins and only bins with at least four cytosines, with each cytosine covered at least four times, with more than 10% more methylation in ZF-SssI than Col-0, and a significance level of less than 0.05 were kept. To define hCG DMRs for T2 and T3, the intersect hCG DMRs of two transgenic lines in each generation were first calculated. Then the union set of T2 and T3 in the same genotype (either ZF-SssI + or ZF-SssI -) were kept. DMRs overlapping with 200 bp bins in each cluster was considered as DMRs specific

for certain clusters. Genomic locations for DMRs and mCG equivalent control were annotated using the Homer 'annotatePeaks' function with default parameters. For T4 and T5, two transgenic lines were separated in order to trace the heritable hCG DMRs. To define heritable hCG DMRs, T2 ZF-SssI – were compared with T2 ZF-SssI + and the shared hCG DMRs were considered as heritable hCG DMRs in T2 ZF-SssI –. For T3 ZF-SssI + and ZF-SssI -, hCG DMRs were overlapped with T2 ZF-SssI +. For T4 ZF-SssI – heritable hCG sites, DMRs were first intersected with T3 ZF-SssI + and then intersected with T2 ZF-SssI +, while T5 ZF-SssI – heritable hCG sites were further intersected with T4 ZF-SssI – hCG DMRs. WGBS data for controls (Col-0 and fwa) used for ZF-SssI in fwa analysis were published before in GSM2932284 38 and GSM3553008 34.

ChIP-seq analysis
For ChIP-seq data, raw reads were first mapped to the reference TAIR10 genome with Bowtie (v0.12.8) by allowing uniquely mapped reads and a maximum of two mismatches. PCR-duplicated reads were then filtered with SAMTools (v 1.19) . To call ZF-SssI FLAG peaks, the MACS2 calldiff function (v 2.1.2) was used to compare ZF-SssI FLAG ChIP-seq and Col-0 FLAG ChIP-seq data with default parameters. Genomic location and enriched motifs of ZF-SssI FLAG-specific peaks were then annotated with Homer 'annotatePeaks' and 'findMotifGenome' functions using 100 bp flanking the summit of the peaks. Promoter regions were defined as default in homer (upstream 1kb and downstream 100bp of TSS). ChIP-seq peaks for histone marks in Col-0 were defined using MACS2 with –nomodel and --call-summits as parameters. ChIP-seq data visualizations were performed using ngs.plot, deepTools, or EnrichedHeatmap.

RNA-seq analysis
For RNA-seq analysis, FastQC was first used to quality-assure the raw reads (v0.11.8). Raw reads were then aligned to the TAIR10 reference genome and TAIR10 gene annotation using STAR (v2.7.0e) with '--outFilterMultimapNmax 1000 --outSAMmultNmax 1' options. Read counts over each gene were then calculated by featureCounts (v2.0.0) with default parameters. Expression levels were determined by RPKM (reads per kilobase of exons per million aligned reads) in R by customized script. Differentially expressed genes were determined with R package DESeq2 using 2 fold change and false discovery rate (FDR) less than 0.05 as cut off. Alternative splicing events were analyzed using rMATS (v4.0.2) with the default parameters. Analysis for DEG associated with hCG DMR in ZF-SssI was performed using the web tool RAD (http://labw.org/rad) with default parameters.

ATAC-seq analysis
ATAC-seq analysis was performed as previously described. Briefly, paired-end reads were aligned to the TAIR10 reference genome with bowtie (v0.12.8) by allowing maximal two mismatches, uniquely mapped reads (-m 1), and the maximal 2 kb distance between pairs (-X 2000). PCR duplicated reads were removed using SAMTools (v1.19) 'rmdup' function and visualized with ngs.plot or deepTools.

For manuscripts utilizing custom algorithms or software that are central to the research but not yet described in published literature, software must be made available to editors and reviewers. We strongly encourage code deposition in a community repository (e.g. GitHub). See the Nature Research guidelines for submitting code & software for further information.

Data

Policy information about availability of data

All manuscripts must include a data availability statement. This statement should provide the following information, where applicable:

- Accession codes, unique identifiers, or web links for publicly available datasets
- A list of figures that have associated raw data
- A description of any restrictions on data availability

The datasets generated and analyzed during the study are available from the corresponding author upon request. All high-throughput sequencing data generated are accessible at NCBI's Gene Expression Omnibus (GEO) via GEO Series accession number GSE158027. WGBS data for controls (Col-0 and fwa) used for ZF-SssI in fwa analysis were published before in GSM2932284 and GSM3553008.

Field-specific reporting

Please select the one below that is the best fit for your research. If you are not sure, read the appropriate sections before making your selection.

☒ Life sciences ☐ Behavioural & social sciences ☐ Ecological, evolutionary & environmental sciences

For a reference copy of the document with all sections, see nature.com/documents/nr-reporting-summary-flat.pdf

Life sciences study design

All studies must disclose on these points even when the disclosure is negative.

Sample size	For flowering time experiments under long day conditions, the size of the sample was chosen based on the standard error of the average of flowering time in Columbia (WT) populations under those conditions.
Data exclusions	No data were excluded.
Replication	RNAseq: We performed RNA-seq in n=4 biological replicates of Col-0 as well as two independent ZF-SssI transgenic lines in both T2 and T3 generations, either with + or without - the transgene (for T2 ZF-SssI + line 1, only three biological replicates were collected; n=35 in total, Table S5).<br>WGBS: We performed WGBS in n=2 biological replicates of Col-0 as well as two independent ZF-SssI transgenic lines in both T2 and T3 generations, either with + or without - the transgene. For T4 and T5, WGBS in n=2 biological replicates of Col-0 and two independent ZF-SssI transgenic lines wihtout the transgene were performed. For ZF-SssI in fwa, n=2 were performed for WGBS data.<br>ChIPseq: ChIPseq were performed in n=1 for WT controls, and n=2 (biological replicates) for ZF-SssI lines. For H2A.Z ChIPseq, additional technical replicates (n=2) were performed.<br>ATACseq: ATACseq were performed in n=1 for WT controls, and n=2 (biological replicates) for ZF-SssI lines.

All attempts at replication were successful for all the experiments mentioned here.

Randomization: For RNAseq, WGBS, ChIPseq and ATACseq, treatment and control samples were grown side by side under same condition.

Blinding: No blinding needed.

Reporting for specific materials, systems and methods

We require information from authors about some types of materials, experimental systems and methods used in many studies. Here, indicate whether each material, system or method listed is relevant to your study. If you are not sure if a list item applies to your research, read the appropriate section before selecting a response.

Materials & experimental systems

n/a	Involved in the study
☐	☒ Antibodies
☒	☐ Eukaryotic cell lines
☒	☐ Palaeontology and archaeology
☒	☐ Animals and other organisms
☒	☐ Human research participants
☒	☐ Clinical data
☒	☐ Dual use research of concern

Methods

n/a	Involved in the study
☐	☒ ChIP-seq
☒	☐ Flow cytometry
☒	☐ MRI-based neuroimaging

Antibodies

Antibodies used:
Anti-FLAG M2 is from Sigma (F1804, 5ul/ChIP used).
Anti-H3K4me1 is from Abcam (Ab8895,20ul/ChIP used).
Anti-H3K4me3 is from Millipore (04-745, 5ul/ChIP used).
Anti-H3K36me3 is from Abcam (Ab9050, 10ul/ChIP used).
Anti-H3K27me3 is from Millipore (07-449, 10ul/ChIP used).
Anti-H3 is from Abcam (Ab1791, (5ul/ChIP used).
Anti-H2A is from Abcam (Ab13923, 10ul/ChIP used).
Anti-PanH3Ac is from Active motif (39140,5ul/ChIP used).
Anti-H2A.Z polyclonal antibodies were raised in rabbits and further purified using the peptides described in Deal, R.B et al, 2007.

Validation:
Validity was tested by comparison of our data with previously published data (anti H2A.Z).
In the case of commercial antibodies validation was carried out by the manufacturer.

Anti-FLAG M2 (https://www.sigmaaldrich.com/technical-documents/articles/biofiles/fluorescence-multiplexing.html)
Anti-H3K4me1 (https://www.abcam.com/histone-h3-mono-methyl-k4-antibody-chip-grade-ab8895.html)
Anti-H3K4me3 (https://www.emdmillipore.com/US/en/product/Anti-trimethyl-Histone-H3-Lys4-Antibody-clone-MC315-rabbit-monoclonal,MM_NF-04-745)
Anti-H3K36me3 (https://www.abcam.com/histone-h3-tri-methyl-k36-antibody-chip-grade-ab9050.html)
Anti-H3K27me3 (https://www.emdmillipore.com/US/en/product/Anti-trimethyl-Histone-H3-Lys27-Antibody,MM_NF-07-449)
Anti-H3 (https://www.abcam.com/histone-h3-antibody-nuclear-marker-and-chip-grade-ab1791.html)
Anti-H2A (https://www.abcam.com/histone-h2a-antibody-chip-grade-ab13923.html)
Anti-PanH3Ac (https://www.activemotif.com/catalog/details/39139/histone-h3ac-pan-acetyl-antibody-pab-1)
Anti-H2A.Z was validated in Potok M E, Wang Y, Xu L, et al. Arabidopsis SWR1-associated protein methyl-CpG-binding domain 9 is required for histone H2A. Z deposition[J]. Nature communications, 2019, 10(1): 1-14.

ChIP-seq

Data deposition

☒ Confirm that both raw and final processed data have been deposited in a public database such as GEO.

☒ Confirm that you have deposited or provided access to graph files (e.g. BED files) for the called peaks.

Data access links
May remain private before publication.
https://www.ncbi.nlm.nih.gov/geo/query/acc.cgi?acc=GSE158027

Files in database submission
GSM4785470 BSPCR_col
GSM4785471 BSPCR_fwa
GSM4785472 BSPCR_fwa_nrpd1
GSM4785473 BSPCR_fwa_nrpe1
GSM4785474 BSPCR_fwa_drm12
GSM4785475 BSPCR_ZFSssI_fwa
GSM4785476 BSPCR_ZFSssI_fwa_nrpd1

GSM4785479 WGBS_col_rep1_for_T2_T3
GSM4785480 WGBS_col_rep2_for_T2_T3
GSM4785481 WGBS_ZFSssI_T2_line1_plus_rep1
GSM4785482 WGBS_ZFSssI_T2_line1_plus_rep2
GSM4785483 WGBS_ZFSssI_T3_line1_plus_rep1
GSM4785484 WGBS_ZFSssI_T3_line1_plus_rep2
GSM4785485 WGBS_ZFSssI_T2_line1_minus_rep1
GSM4785486 WGBS_ZFSssI_T2_line1_minus_rep2
GSM4785487 WGBS_ZFSssI_T3_line1_minus_rep1
GSM4785488 WGBS_ZFSssI_T3_line1_minus_rep2
GSM4785489 WGBS_ZFSssI_T2_line2_plus_rep1
GSM4785490 WGBS_ZFSssI_T2_line2_plus_rep2
GSM4785491 WGBS_ZFSssI_T3_line2_plus_rep1
GSM4785492 WGBS_ZFSssI_T3_line2_plus_rep2
GSM4785493 WGBS_ZFSssI_T2_line2_minus_rep1
GSM4785494 WGBS_ZFSssI_T2_line2_minus_rep2
GSM4785495 WGBS_ZFSssI_T3_line2_minus_rep1
GSM4785496 WGBS_ZFSssI_T3_line2_minus_rep2
GSM4785497 WGBS_col_rep1_for_T4
GSM4785498 WGBS_col_rep2_for_T4
GSM4785499 WGBS_ZFSssI_T4_line1_minus_rep1
GSM4785500 WGBS_ZFSssI_T4_line1_minus_rep2
GSM4785501 WGBS_ZFSssI_T4_line2_minus_rep1
GSM4785502 WGBS_ZFSssI_T4_line2_minus_rep2
GSM4785503 WGBS_col_rep1_for_T5_part1
GSM4785504 WGBS_col_rep2_for_T5_part1
GSM4785505 WGBS_ZFSssI_T5_line1_minus_rep1_part1
GSM4785506 WGBS_ZFSssI_T5_line1_minus_rep2_part1
GSM4785507 WGBS_ZFSssI_T5_line2_minus_rep1_part1
GSM4785508 WGBS_ZFSssI_T5_line2_minus_rep2_part1
GSM4785509 WGBS_ZFSssI_in_fwa_line1
GSM4785510 WGBS_ZFSssI_in_fwa_line2
GSM4785511 RNAseq_col_for_T2_T3_rep1
GSM4785512 RNAseq_col_for_T2_T3_rep2
GSM4785513 RNAseq_col_for_T2_T3_rep3
GSM4785514 RNAseq_col_for_T2_T3_rep4
GSM4785515 RNAseq_ZFSssI_T2_line2_minus_rep1
GSM4785516 RNAseq_ZFSssI_T2_line2_minus_rep2
GSM4785517 RNAseq_ZFSssI_T2_line2_minus_rep3
GSM4785518 RNAseq_ZFSssI_T2_line2_minus_rep4
GSM4785519 RNAseq_ZFSssI_T2_line1_minus_rep1
GSM4785520 RNAseq_ZFSssI_T2_line1_minus_rep2
GSM4785521 RNAseq_ZFSssI_T2_line1_minus_rep3
GSM4785522 RNAseq_ZFSssI_T2_line1_minus_rep4
GSM4785523 RNAseq_ZFSssI_T3_line2_minus_rep1
GSM4785524 RNAseq_ZFSssI_T3_line2_minus_rep2
GSM4785525 RNAseq_ZFSssI_T3_line2_minus_rep3
GSM4785526 RNAseq_ZFSssI_T3_line2_minus_rep4
GSM4785527 RNAseq_ZFSssI_T3_line1_minus_rep1
GSM4785528 RNAseq_ZFSssI_T3_line1_minus_rep2
GSM4785529 RNAseq_ZFSssI_T3_line1_minus_rep3
GSM4785530 RNAseq_ZFSssI_T3_line1_minus_rep4
GSM4785531 RNAseq_ZFSssI_T2_line2_plus_rep1
GSM4785532 RNAseq_ZFSssI_T2_line2_plus_rep2
GSM4785533 RNAseq_ZFSssI_T2_line2_plus_rep3
GSM4785534 RNAseq_ZFSssI_T2_line2_plus_rep4
GSM4785535 RNAseq_ZFSssI_T3_line2_plus_rep1
GSM4785536 RNAseq_ZFSssI_T3_line2_plus_rep2
GSM4785537 RNAseq_ZFSssI_T3_line2_plus_rep3
GSM4785538 RNAseq_ZFSssI_T3_line2_plus_rep4
GSM4785539 RNAseq_ZFSssI_T2_line1_plus_rep1
GSM4785540 RNAseq_ZFSssI_T2_line1_plus_rep2
GSM4785541 RNAseq_ZFSssI_T2_line1_plus_rep3
GSM4785542 RNAseq_ZFSssI_T3_line1_plus_rep1
GSM4785543 RNAseq_ZFSssI_T3_line1_plus_rep2
GSM4785544 RNAseq_ZFSssI_T3_line1_plus_rep3
GSM4785545 RNAseq_ZFSssI_T3_line1_plus_rep4
GSM4785546 ChIP_FLAG_col
GSM4785547 ChIP_FLAG_ZFSssI_line1
GSM4785548 ChIP_FLAG_ZFSssI_line2
GSM4785549 ChIP_H3K4me1_col
GSM4785550 ChIP_H3K4me1_ZFSssI_line1
GSM4785551 ChIP_H3K4me1_ZFSssI_line2
GSM4785552 ChIP_H3K4me3_col
GSM4785553 ChIP_H3K4me3_ZFSssI_line1
GSM4785554 ChIP_H3K4me3_ZFSssI_line2

GSM4785555 ChIP_H3K36me3_col
GSM4785556 ChIP_H3K36me3_ZFSssI_line1
GSM4785557 ChIP_H3K36me3_ZFSssI_line2
GSM4785558 ChIP_H3_col
GSM4785559 ChIP_H3_ZFSssI_line1
GSM4785560 ChIP_H3_ZFSssI_line2
GSM4785561 ChIP_H2A_col
GSM4785562 ChIP_H2A_ZFSssI_line1_rep1
GSM4785563 ChIP_H2A_ZFSssI_line2_rep1
GSM4785564 ChIP_H2AZ_col_rep1
GSM4785565 ChIP_H2AZ_ZFSssI_line1
GSM4785566 ChIP_H2AZ_ZFSssI_line2
GSM4785567 ChIP_PanH3Ac_col
GSM4785568 ChIP_PanH3Ac_ZFSssI_line1
GSM4785569 ChIP_PanH3Ac_ZFSssI_line2
GSM4785570 ChIP_H2AZ_col_rep2
GSM4785571 ChIP_H2AZ_ZFSssI_line1_rep2
GSM4785572 ChIP_H2AZ_ZFSssI_line2_rep2
GSM4785573 ChIP_H3K27me3_col
GSM4785574 ChIP_H3K27me3_ZFSssI_line1
GSM4785575 ChIP_H3K27me3_ZFSssI_line2
GSM4785576 ATACseq_col
GSM4785577 ATACseq_ZFSssI_line1
GSM4785578 ATACseq_ZFSssI_line2
GSM4955357 BSPCR_ZFSssI_fwa_nrpe1
GSM4955358 BSPCR_ZFSss_fwa_drm12

Genome browser session
(e.g. UCSC)

N.A.

Methodology

Replicates

ChIPseq: ChIPseq were performed in n=1 for WT controls, and n=2 (biological replicates) for ZF-SssI lines. For H2A.Z ChIPseq, additional technical replicates (n=2) were performed.

Sequencing depth

Samplle ID Sample IP Genotype Raw reads Aligned reads PCR duplicates
1 ChIP_FLAG_col FLAG Col-0 22729083 16439270(72.33%) 5185999(22.82%)
2 ChIP_FLAG_ZFSssI_line1 FLAG ZF-SssI line 1 13622389 9899176(72.67%) 2821658(20.71%)
3 ChIP_FLAG_ZFSssI_line2 FLAG ZF-SssI line 2 25703892 19091374(74.27%) 5673988(22.07%)
4 ChIP_H3K4me1_col H3K4me1 Col-0 7467463 6900245(92.40%) 415147(5.56%)
5 ChIP_H3K4me1_ZFSssI_line1 H3K4me1 ZF-SssI line 1 9146794 8489917(92.82%) 501942(5.49%)
6 ChIP_H3K4me1_ZFSssI_line2 H3K4me1 ZF-SssI line 2 9850655 9059942(91.97%) 543377(5.52%)
7 ChIP_H3K4me3_col H3K4me3 Col-0 9243294 8586615(92.90%) 395022(4.27%)
8 ChIP_H3K4me3_ZFSssI_line1 H3K4me3 ZF-SssI line 1 7828187 7337096(93.73%) 326367(4.17%)
9 ChIP_H3K4me3_ZFSssI_line2 H3K4me3 ZF-SssI line 2 10687696 9881298(92.45%) 583679(5.46%)
10 ChIP_H3K36me3_col H3K36me3 Col-0 18347841 15387630(83.87%) 2503311(13.64%)
11 ChIP_H3K36me3_ZFSssI_line1 H3K36me3 ZF-SssI line 1 15790507 13088146(82.89%) 2264179(14.34%)
12 ChIP_H3K36me3_ZFSssI_line2 H3K36me3 ZF-SssI line 2 12607182 9467665(75.10%) 1771816(14.05%)
13 ChIP_H3_col H3 Col-0 9580553 6613559(69.03%) 2537443(26.49%)
14 ChIP_H3_ZFSssI_line1 H3 ZF-SssI line 1 10456173 7453102(71.28%) 2709847(25.92%)
15 ChIP_H3_ZFSssI_line2 H3 ZF-SssI line 2 15396354 10728334(69.68%) 4247029(27.58%)
16 ChIP_H2A_col H2A Col-0 19210385 15253078(79.40%) 3314823(17.26%)
17 ChIP_H2A_ZFSssI_line1_rep1 H2A ZF-SssI line 1 14937757 11930494(79.87%) 2543683(17.03%)
18 ChIP_H2A_ZFSssI_line2_rep1 H2A ZF-SssI line 2 19888168 15374161(77.30%) 3840546(19.31%)
19 ChIP_H2AZ_col_rep1 H2AZ Col-0 8086839 7377145(91.22%) 592112(7.32%)
20 ChIP_H2AZ_ZFSssI_line1 H2AZ ZF-SssI line 1 9313007 8459846(90.84%) 697447(7.49%)
21 ChIP_H2AZ_ZFSssI_line2 H2AZ ZF-SssI line 2 11190405 9986455(89.24%) 813263(7.27%)
22 ChIP_PanH3Ac_col PanH3Ac Col-0 18361292 14971988(81.54%) 2914599(15.87%)
23 ChIP_PanH3Ac_ZFSssI_line1 PanH3Ac ZF-SssI line 1 19348497 15664063(80.96%) 3204007(16.56%)
24 ChIP_PanH3Ac_ZFSssI_line2 PanH3Ac ZF-SssI line 2 24023206 20399766(84.92%) 3029661(12.61%)
25 ChIP_H2AZ_col_rep2 H2AZ Col-0 33673791 16129089 (47.90%) 5958014(18.47%)
26 ChIP_H2AZ_ZFSssI_line1_rep2 H2AZ ZF-SssI line 1 34703442 16637538 (47.94%) 6421180(19.30%)
27 ChIP_H2AZ_ZFSssI_line2_rep2 H2AZ ZF-SssI line 2 41989085 18314534 (43.62%) 7349676(20.07%)
28 ChIP_H3K27me3_col H3K27me3 Col-0 24134085 11495781(47.63%) 5044483(21.94%)
29 ChIP_H3K27me3_ZFSssI_line1 H3K27me3 ZF-SssI line 1 28310214 13659781 (48.25%) 6392982(23.40%)
30 ChIP_H3K27me3_ZFSssI_line2 H3K27me3 ZF-SssI line 2 21243174 9366946 (44.09%) 3595296(19.19%)

Antibodies

Anti-FLAG M2 (F1804, Sigma), H3K4me1 (Ab8895, Abcam), H3K4me3 (04-745, Millipore), H3K36me3 (Ab9050, Abcam), H3K27me3 (07-449, Millipore), H3 (Ab1791, Abcam), H2A (Ab13923, Abcam), H2A.Z 63, and PanH3Ac (39140, Active motif).

Peak calling parameters

To call ZF-SssI FLAG peaks, the MACS2 calldiff function 70 (v 2.1.2) was used to compare ZF-SssI FLAG ChIP-seq and Col-0 FLAG ChIP-seq data with default parameters.

Data quality

Since we are comparing the FLAG ChIPseq in ZF-SssI lines and Col-0 FLAG ChIPseq data, instead of calling ChIPseq peaks vs input, we used MACS2 calldiff function to call the differential binding events in ZF-SssI FLAG ChIPseq. We identified 2151 differential binding

events in ZF-SssI FLAG compared to Col-0 FLAG ChIP-seq.

Software

Bowtie
SAMTools
MACS2 calldiff function
Homer
ngs.plot
deepTools
EnrichedHeatmap
